# Supplementary material for: Synthesis and Antimicrobial Properties of a Ciprofloxacin and PAMAM-dendrimer Conjugate
Source: Molecules. 2020 Mar 18;25(6):1389. doi: 10.3390/molecules25061389 (PMC7146445; doi:10.3390/molecules25061389)
Supplement: Supplementary file 1 [file molecules-25-01389-s001.pdf]

Supplementary material for:

Synthesis and Antimicrobial Properties of a Ciprofloxacin and PAMAM-dendrimer Conjugate.

Søren Wedel Svenningsen,[1] Rikki Franklin Frederiksen,[2] Claire Counil,[1] Mario Ficker,[1] Jørgen J. Leisner,[2] and Jørn B. Christensen[1*]

[1] Department of Chemistry, University of Copenhagen, Thorvaldsensvej 40, DK-1871 Frederiksberg C, Denmark; swsvenningsen@gmail.com (SWS); claire.counil@orange.fr (CC); mario.ficker@gmail.com (MF); jbc@chem.ku.dk (JBC)

[2] Department of Veterinary and Animal Sciences, Food Safety and Zoonoses, University of Copenhagen, Grønnegårdsvej 15, DK-1870 Frederiksberg C, Denmark; rikki.fredriksen@ki.se (RFF); jjl@sund.ku.dk (JL)

* Correspondence: jbc@chem.ku.dk; Tel.: +45-3533-2452

Table of content:

S1. Optical Densities at 600 nm versus well number for the strains 4/74 and PAO1.

S2 – S5	NMR-spectra of compound 2
S6 – S9	NMR-spectra of compound 3
S10 – S14	ESI-MS spectra of compound 3
S15 – S19	NMR-spectra of compound 4
S20 – S22	ESI-MS spectra of compound 4
S23 – S26	NMR-spectra of compound 5
S27 – S28	ESI-MS spectra of compound 5
S29 – S32	NMR-spectra of compound 7
S33 – S34	ESI-MS spectra of compound 7
S35 – S39	NMR-spectra of compound 8
S40	ESI-MS spectrum of compound 8
S41 – S45	NMR-spectra of compound 9
S46 – S49	ESI-MS spectra of compound 9
S50 – S54	NMR-spectra of compound 10
S55 – S57	ESI-MS spectra of compound 10

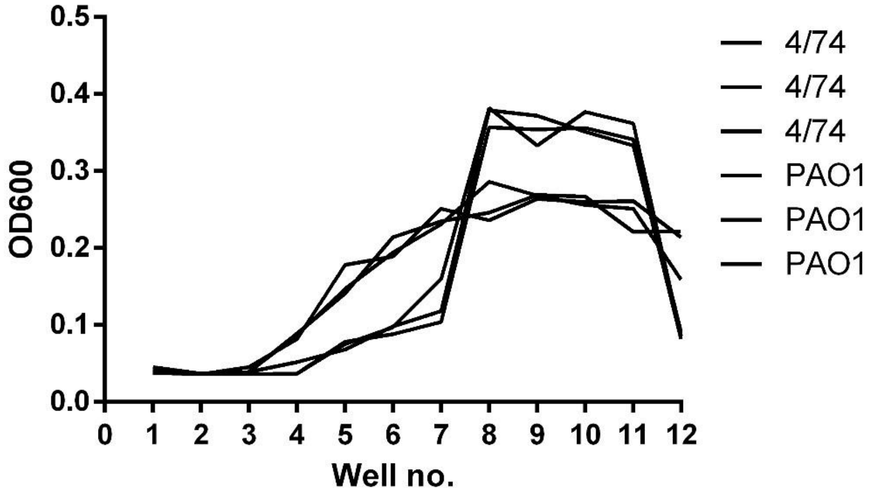

Figure S1: Optical Densities at 600 nm versus well number for the strains 4/74 and PAO1.

Figure S2: ^{1}H-NMR (500 MHz in DMSO-D$_6$) of DAB-PAMAM-G0-$(NH_2)_4$ (**2**)

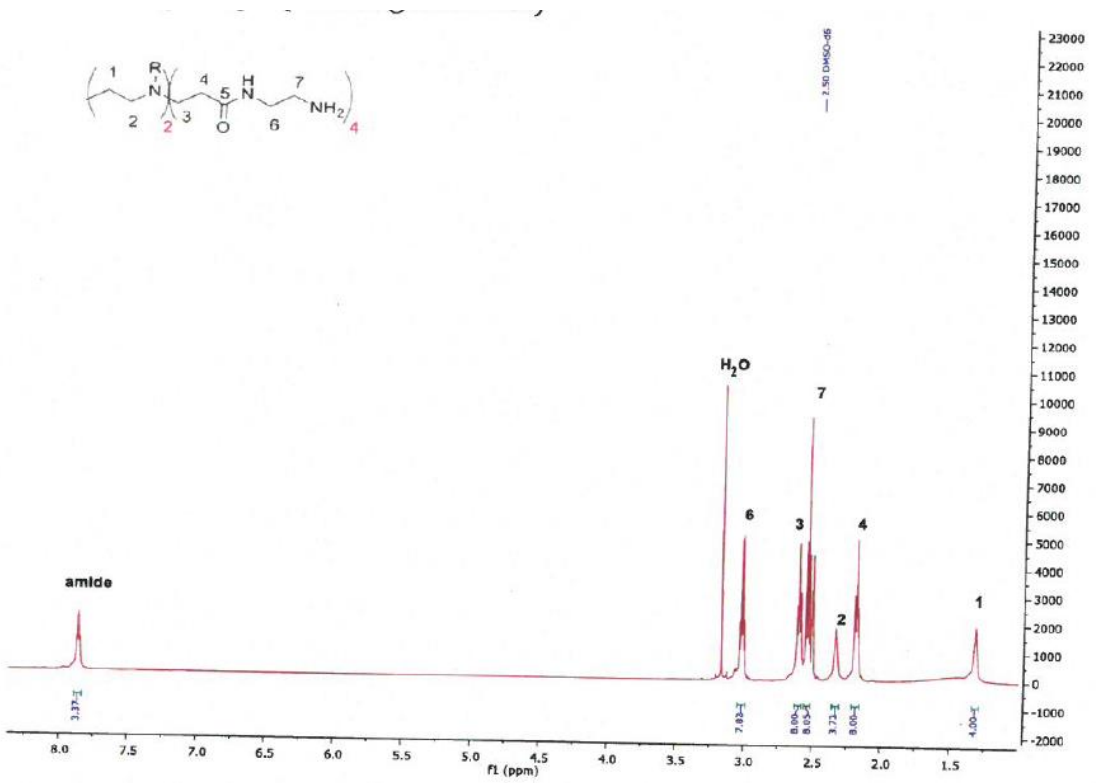

Figure S3: COSY-NMR (500 MHz in DMSO-D_6) of DAB-PAMAM-G0-$(NH_2)_4$ (**2**)

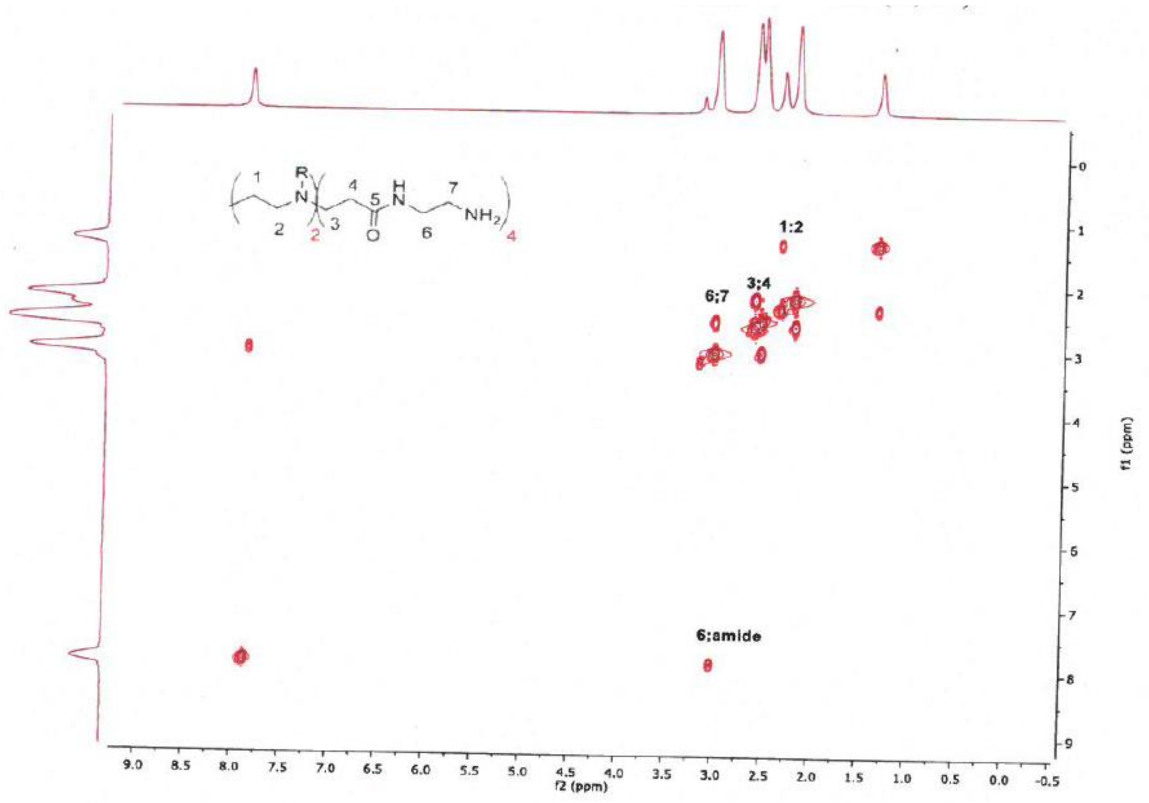

Figure S4: ^{13}C-NMR (125 MHz in DMSO-D_6) of DAB-PAMAM-G0-$(NH_2)_4$ (**2**)

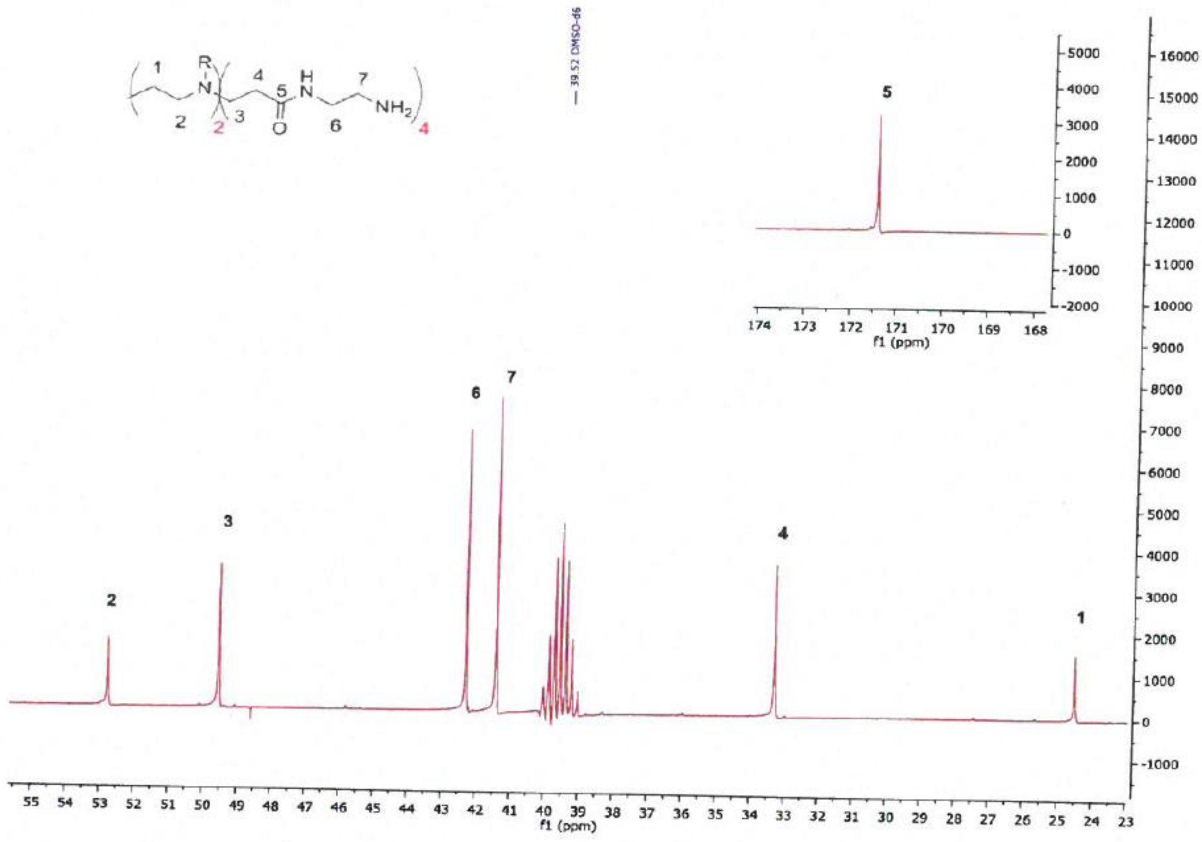

Figure S5: HSQC-NMR (125 & 500 MHz in DMSO-D$_6$) of DAB-PAMAM-G0-(NH$_2$)$_4$ (**2**)

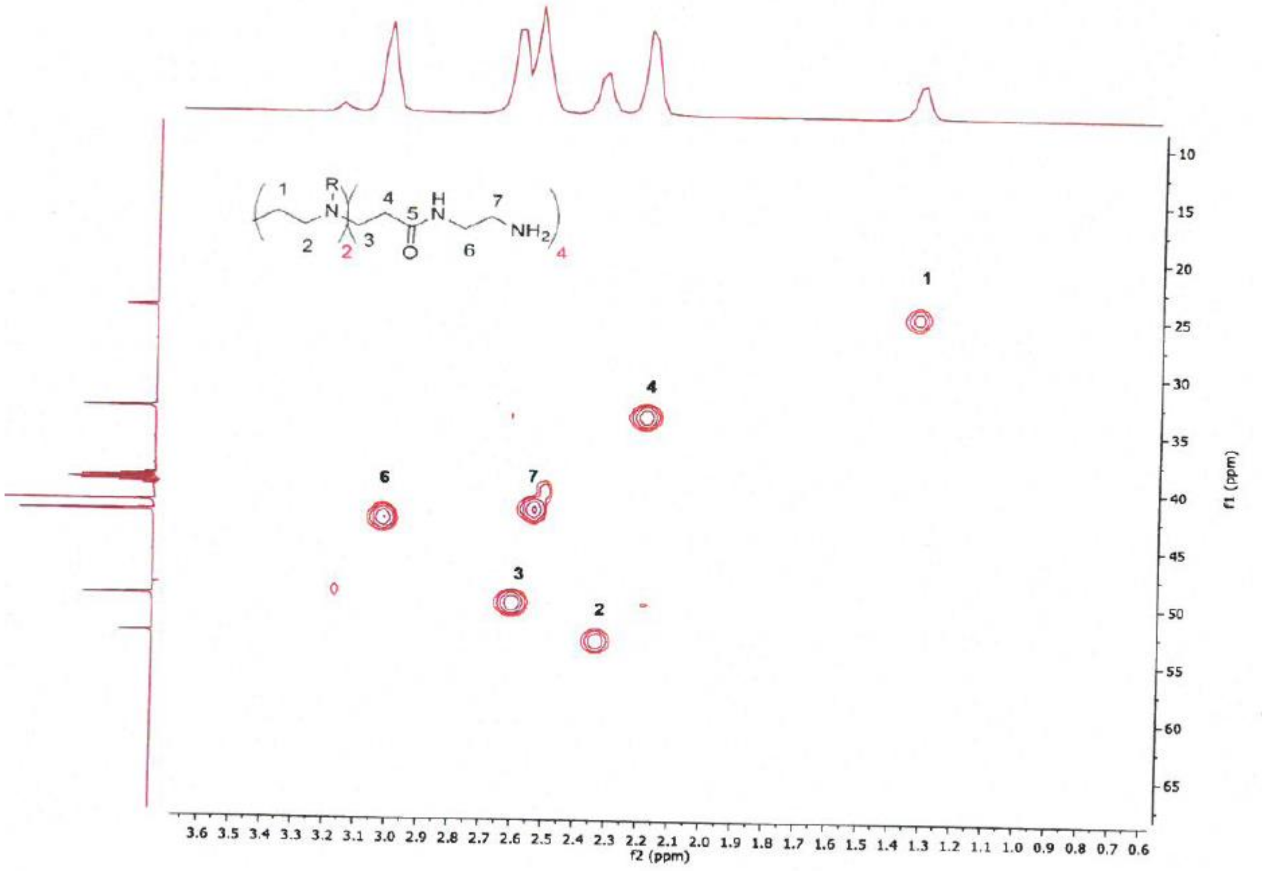

Figure S6: ^{1}H-NMR (500 MHz in DMSO-D$_6$) of Compound **3**.

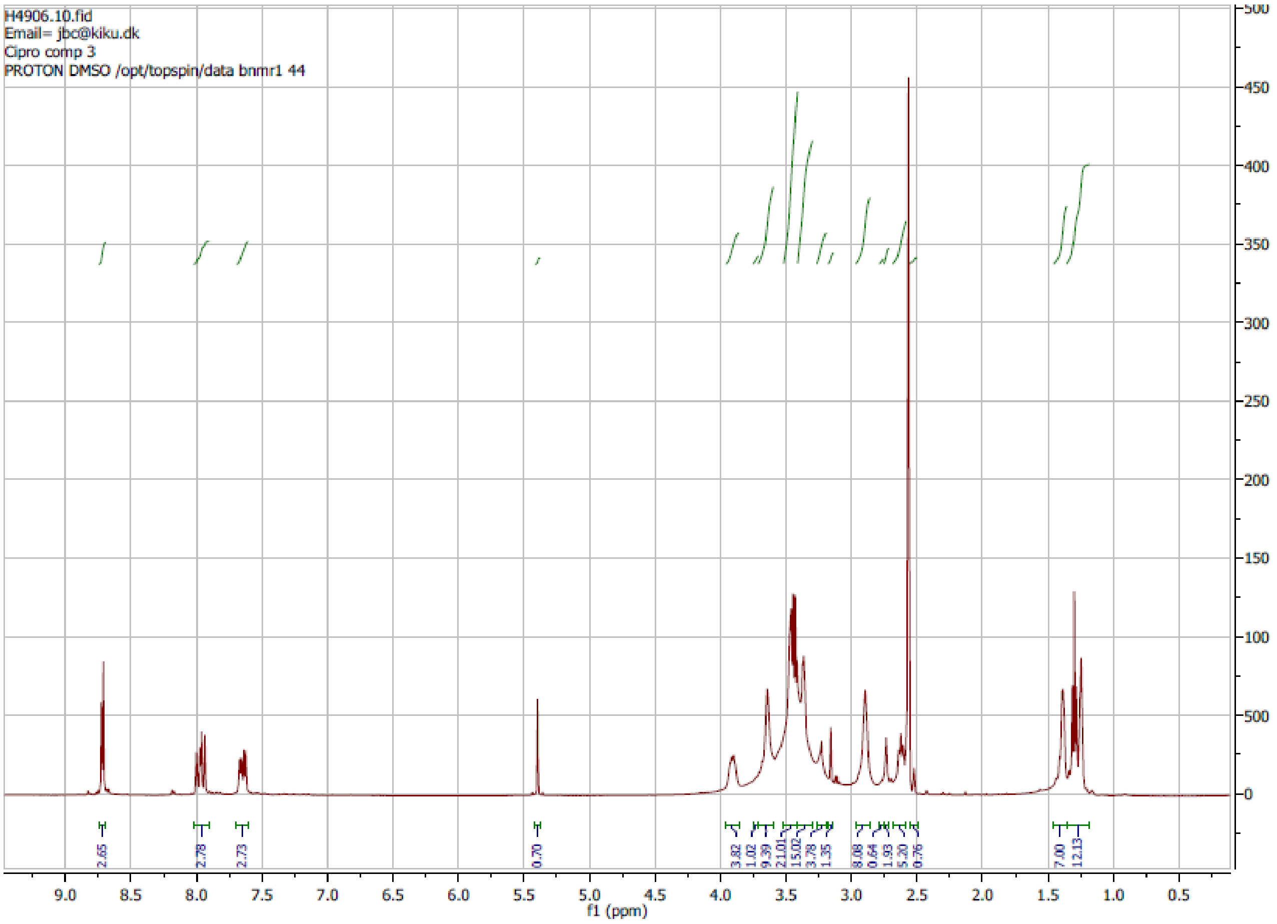

Figure S7: ^{13}C-NMR (125 MHz in DMSO-D_6) of Compound **3**.

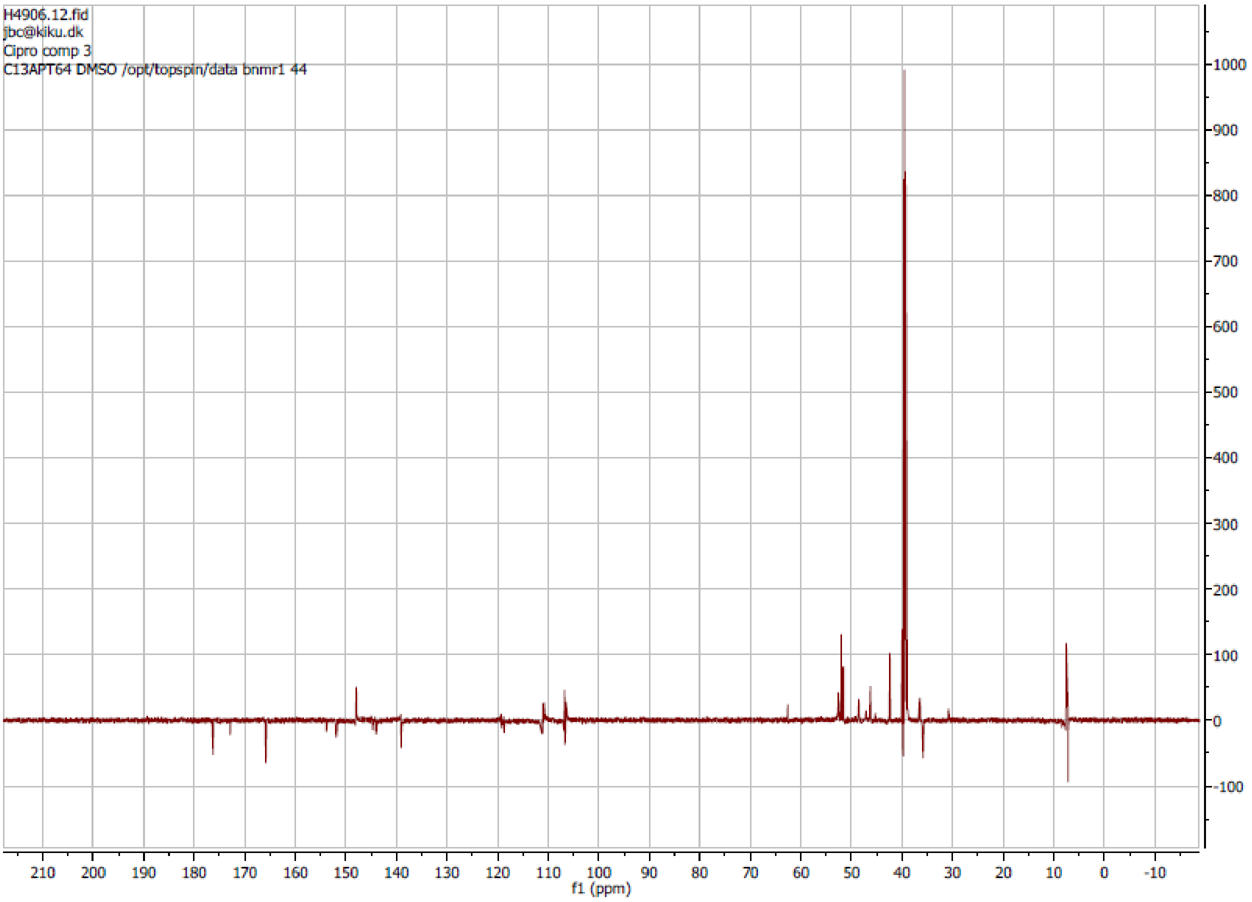

Figure S8: COSY-NMR (500 MHz in DMSO-D$_6$) of Compound **3**.

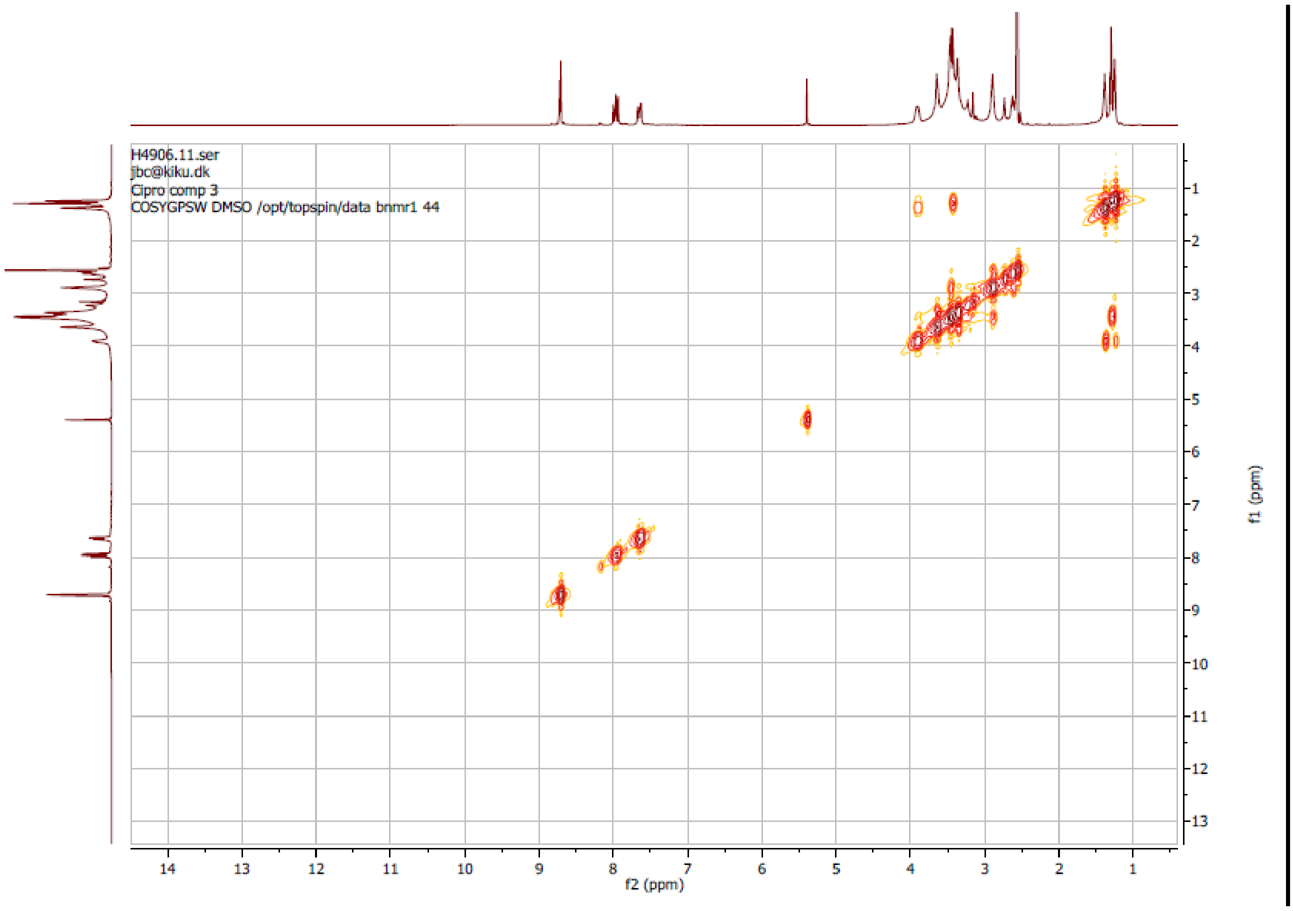

Figure S9: HSQC-NMR (125 & 500 MHz in DMSO-D_6) of Compound **3**

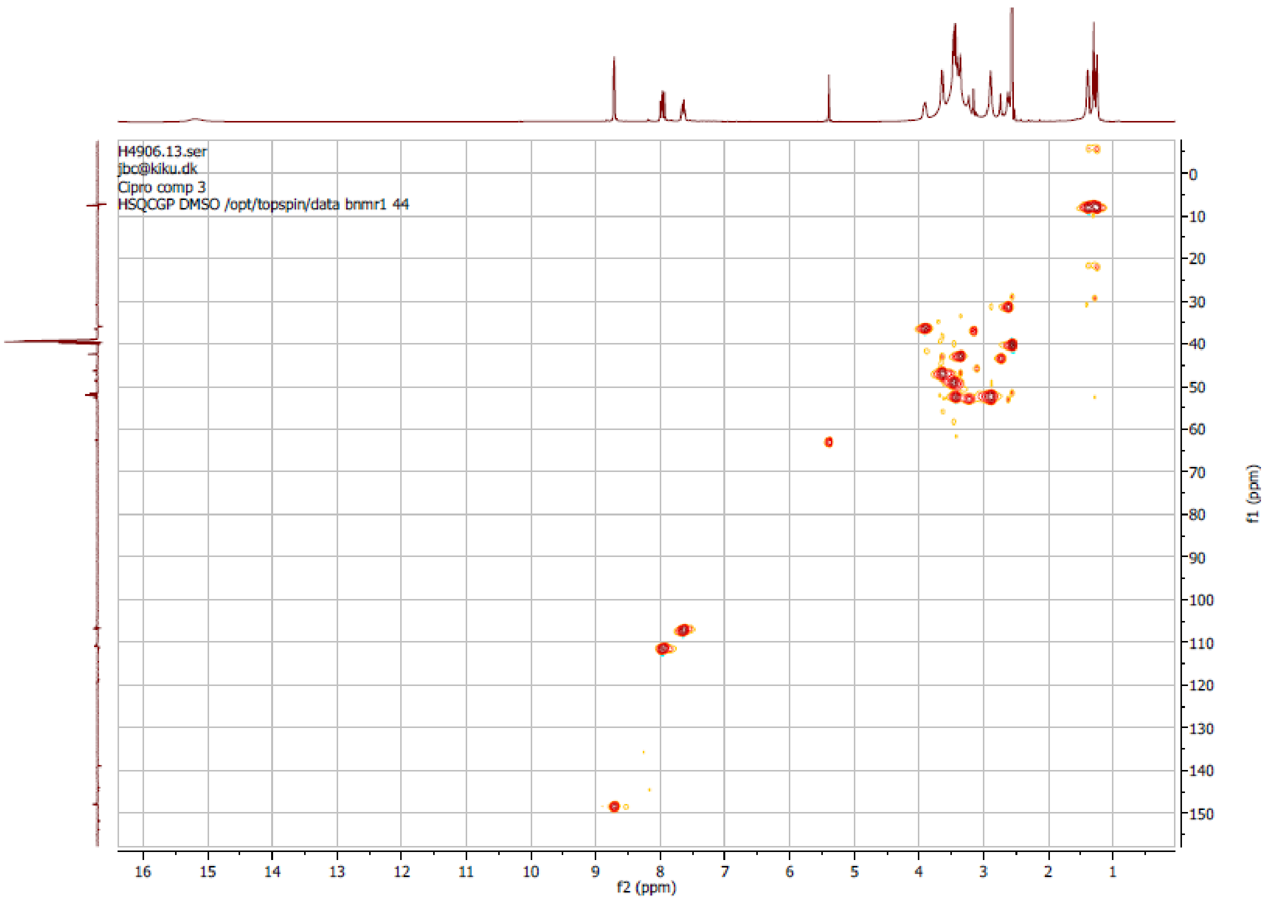

Figure S10: ES-MS of Compound **3**

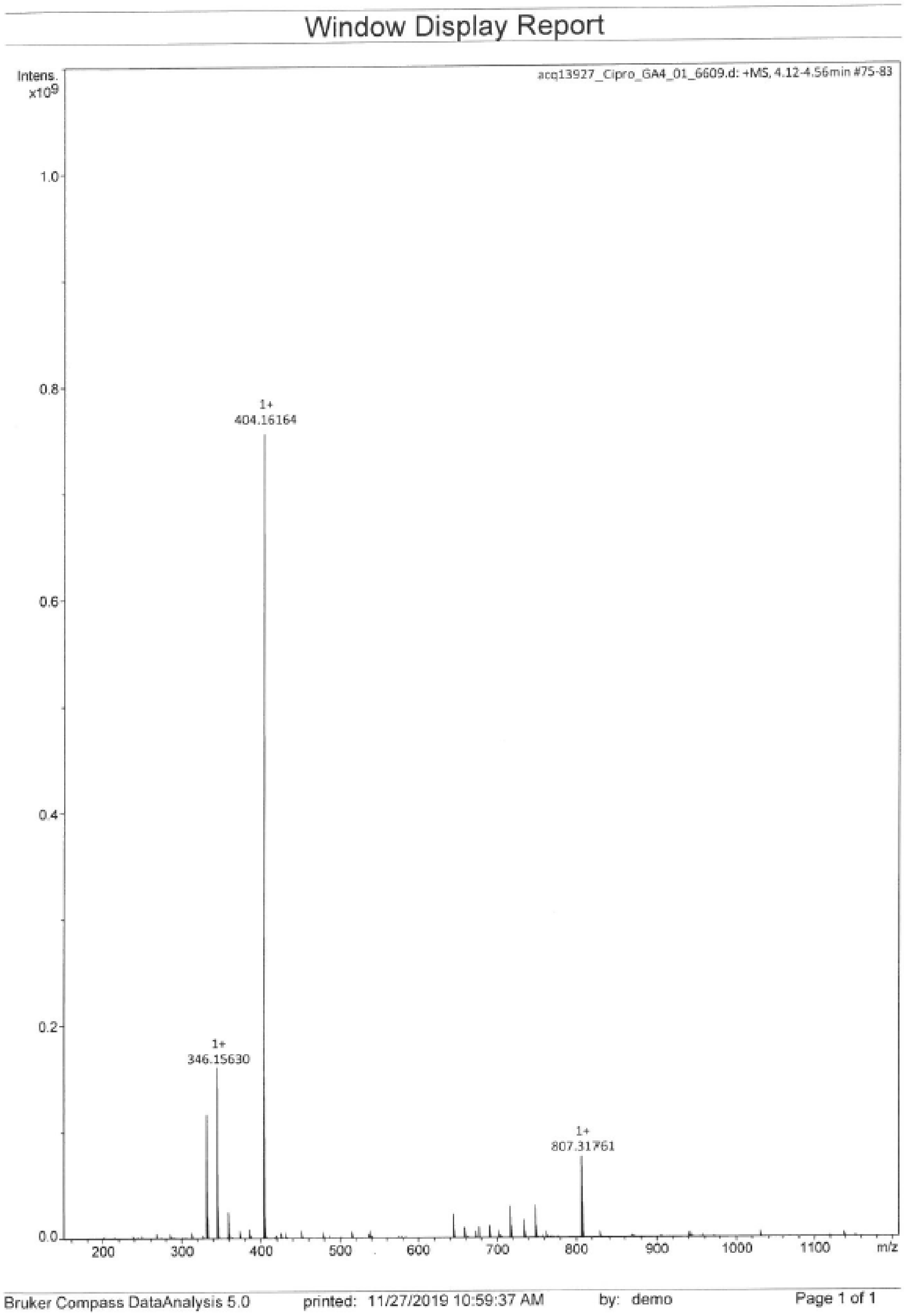

Figure S11: ES-MS of Compound **3**

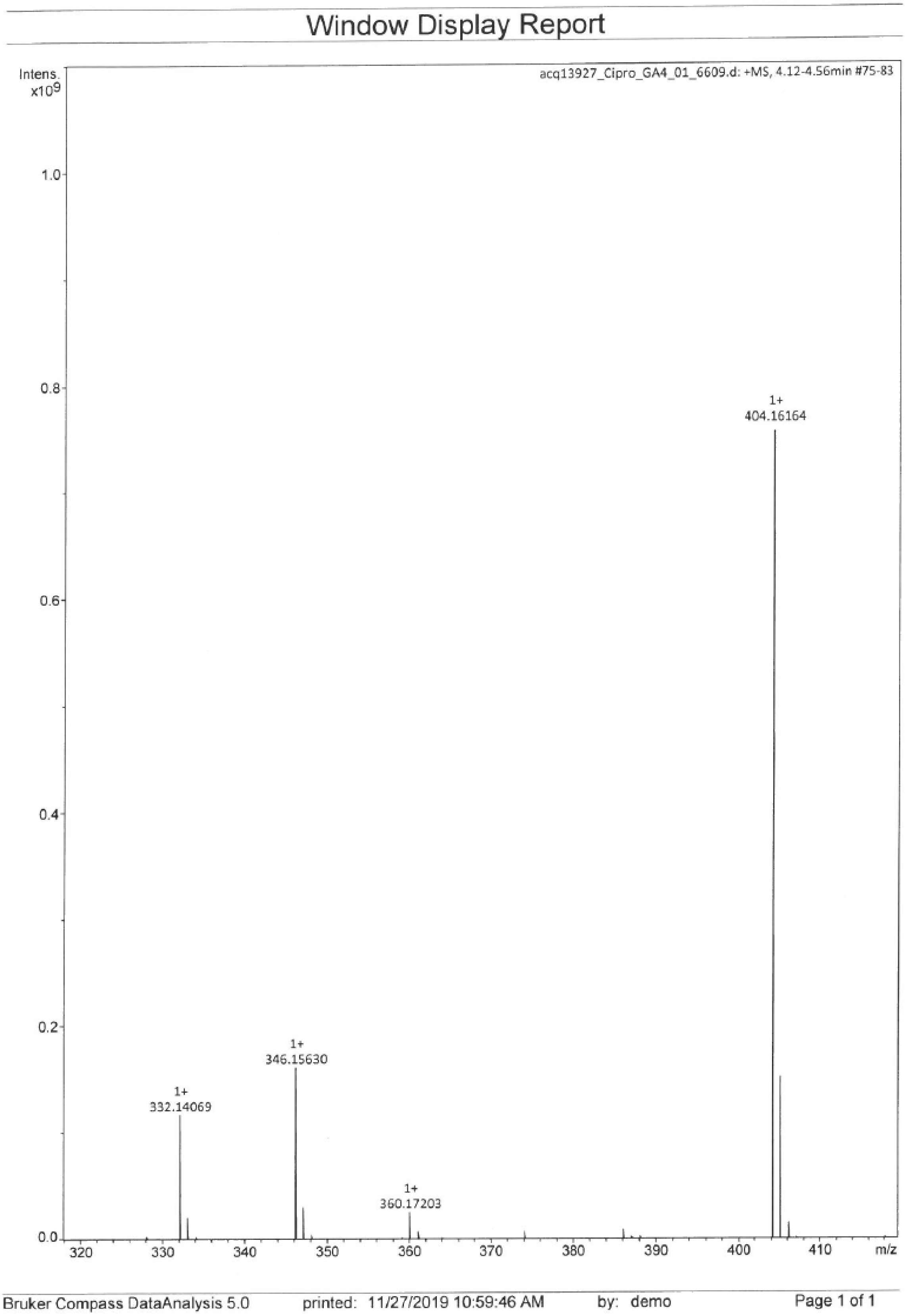

Figure S12: ES-MS of Compound **3**

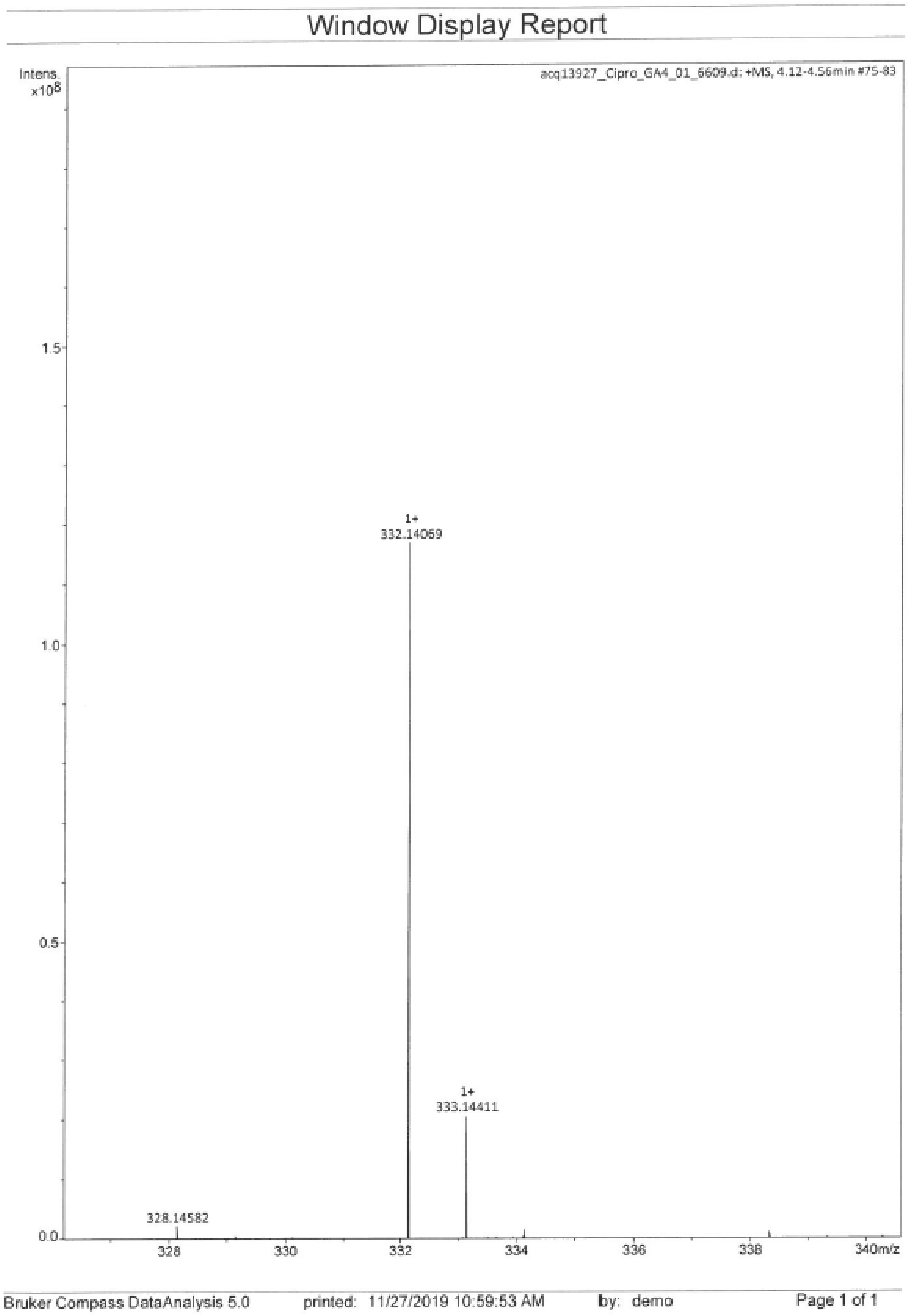

Figure S13: ES-MS of Compound **3**

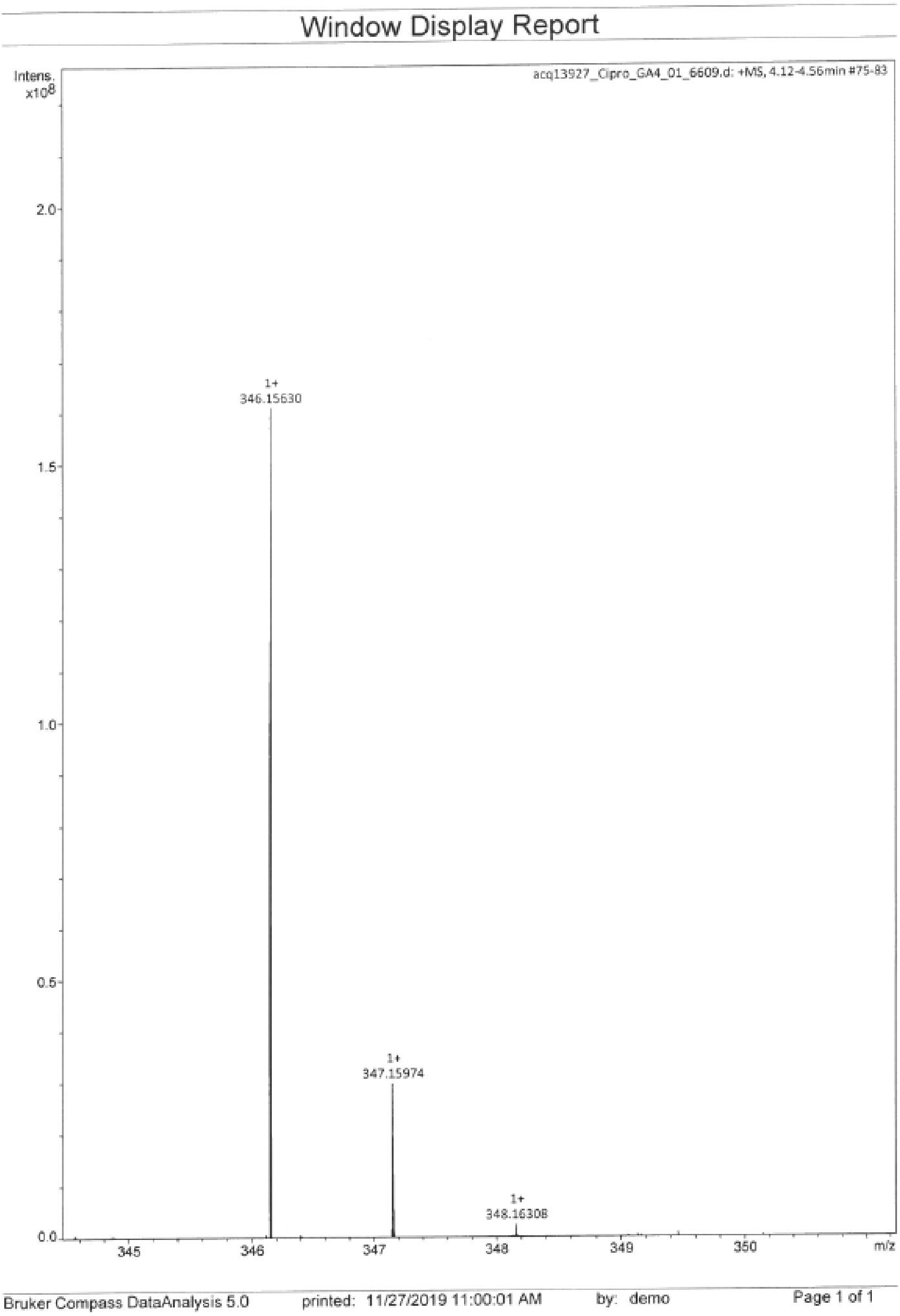

Figure S14: ES-MS of Compound **3**

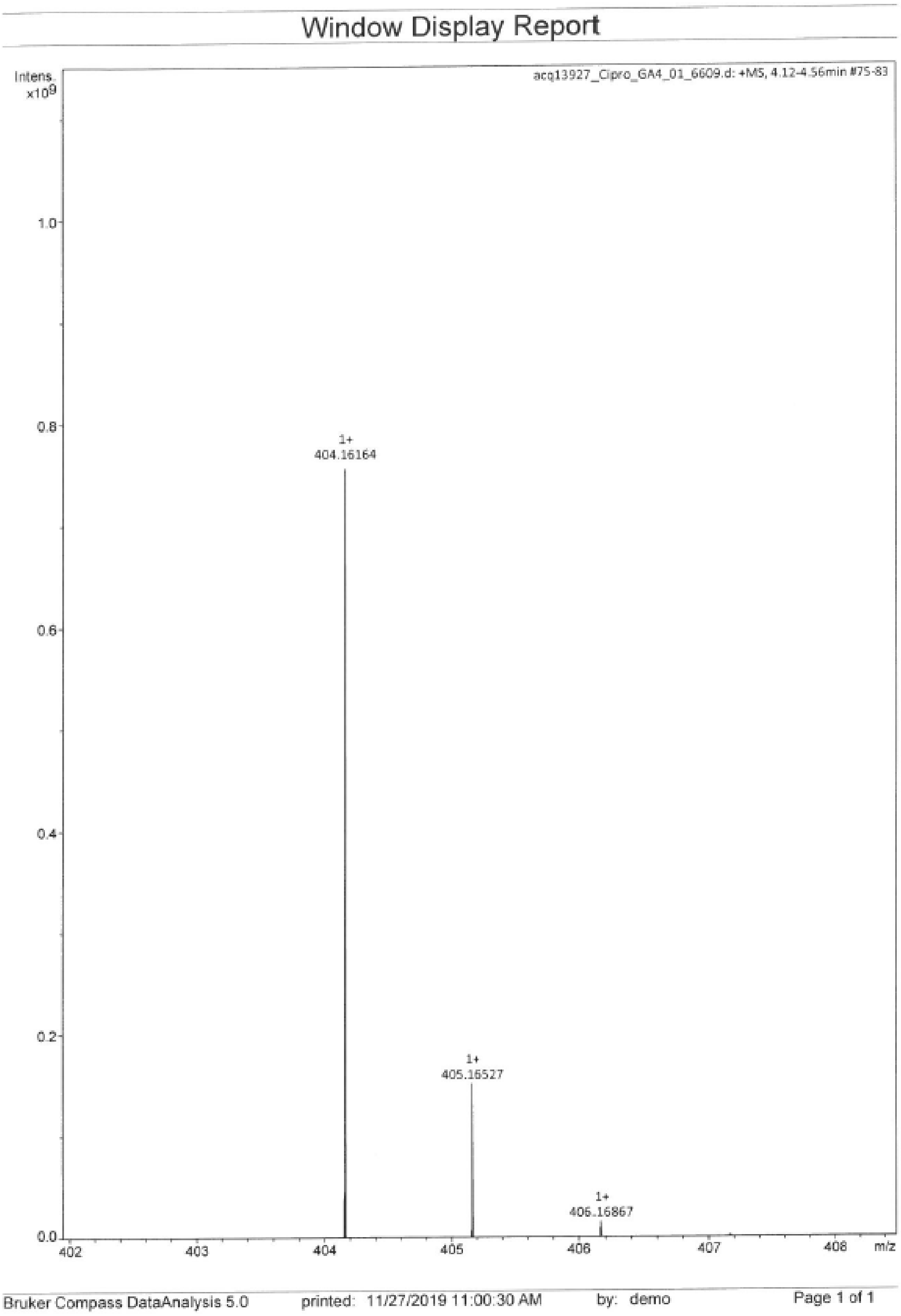

Figure S15: ^{1}H-NMR (500 MHz in DMSO-D_6) of **DAB-PAMAM-G0-(Cipro-COOH)$_4$ • 6HCl (4)**

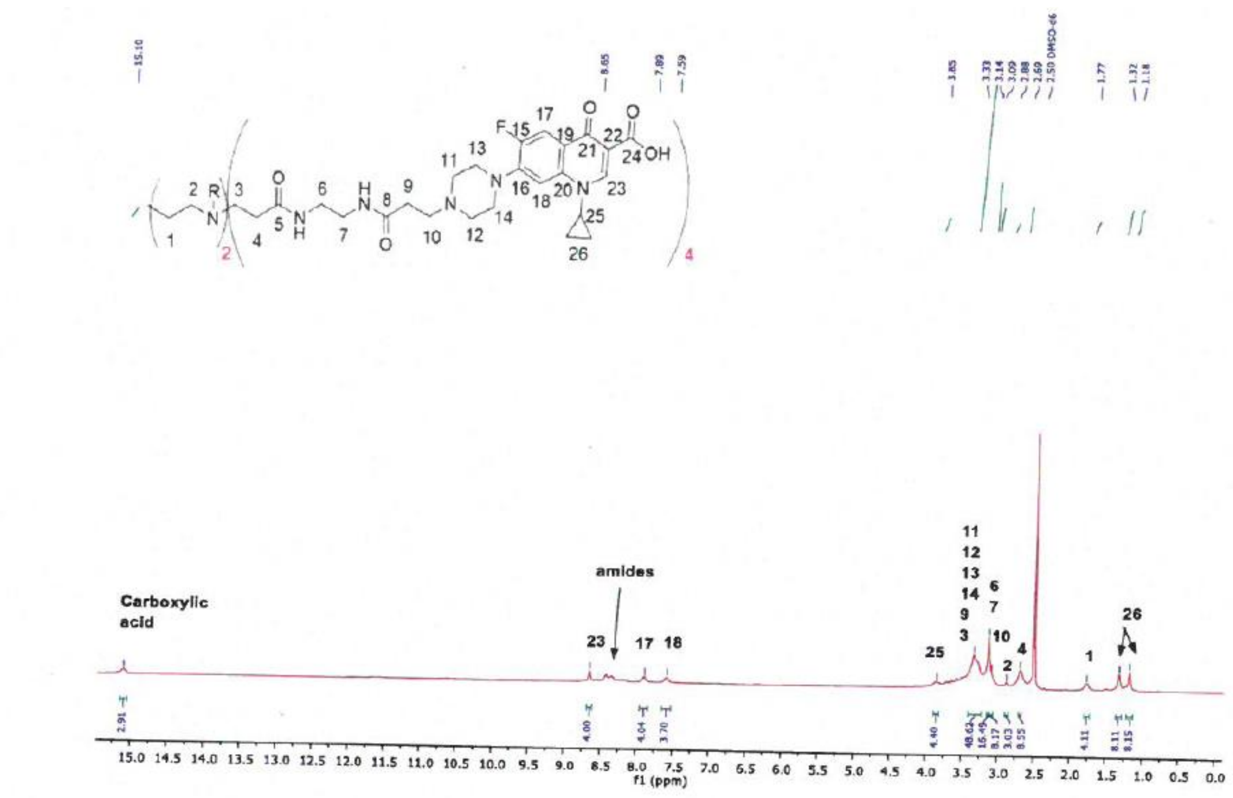

Figure S16: ^{13}C-NMR (126 MHz in DMSO-d6) of **DAB-PAMAM-G0-(Cipro-COOH)$_4$ • 6HCl (4).**

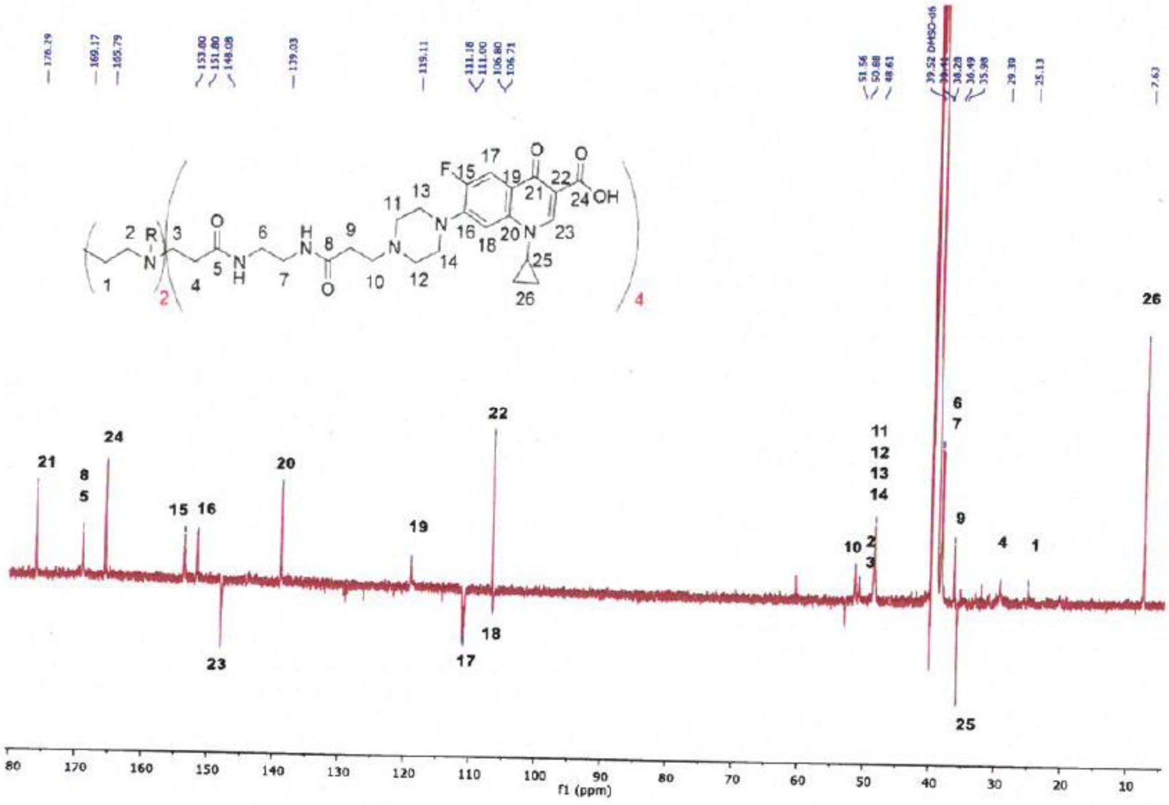

Figure S17: COSY-NMR (500 MHz in DMSO-D$_6$) of **DAB-PAMAM-G0-(Cipro-COOH)$_4$ • 6HCl (4).**

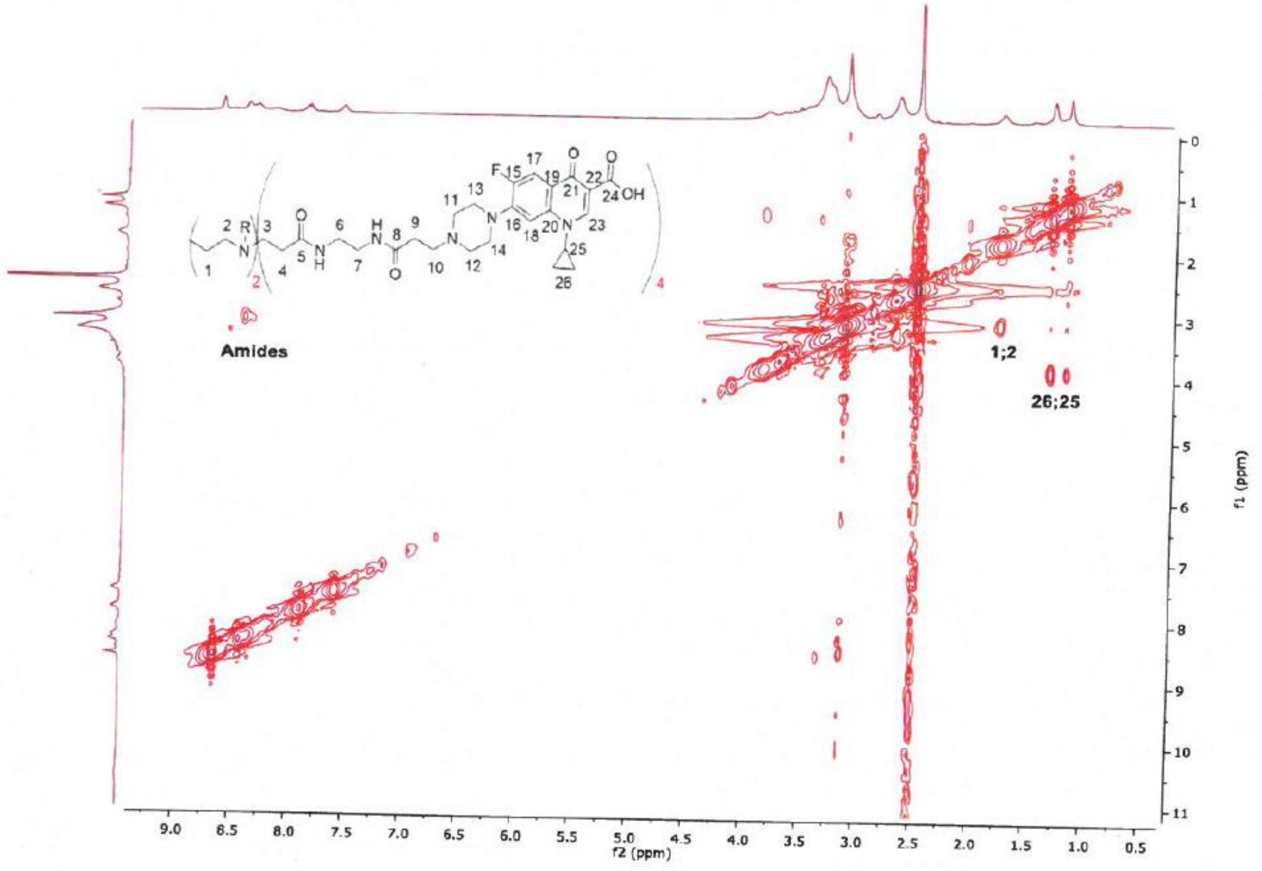

Figure S18: HSQC-NMR (125 & 500 MHz in DMSO-D_6) of **DAB-PAMAM-G0-(Cipro-COOH)$_4$ • 6HCl (4).**

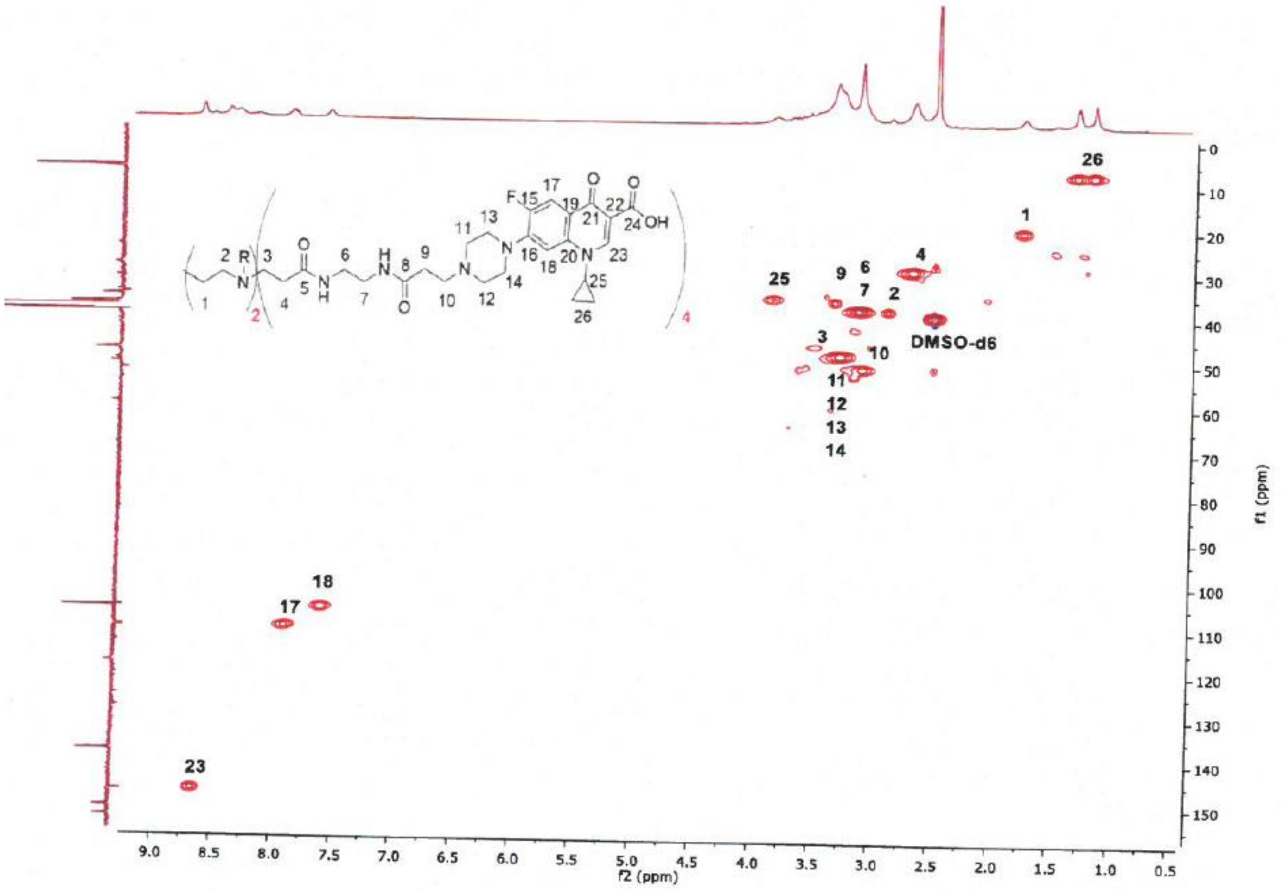

Figure S19: ^{19}F-NMR (470 MHz in DMSO-D$_6$) of **DAB-PAMAM-G0-(Cipro-COOH)$_4$ • 6HCl (4).**

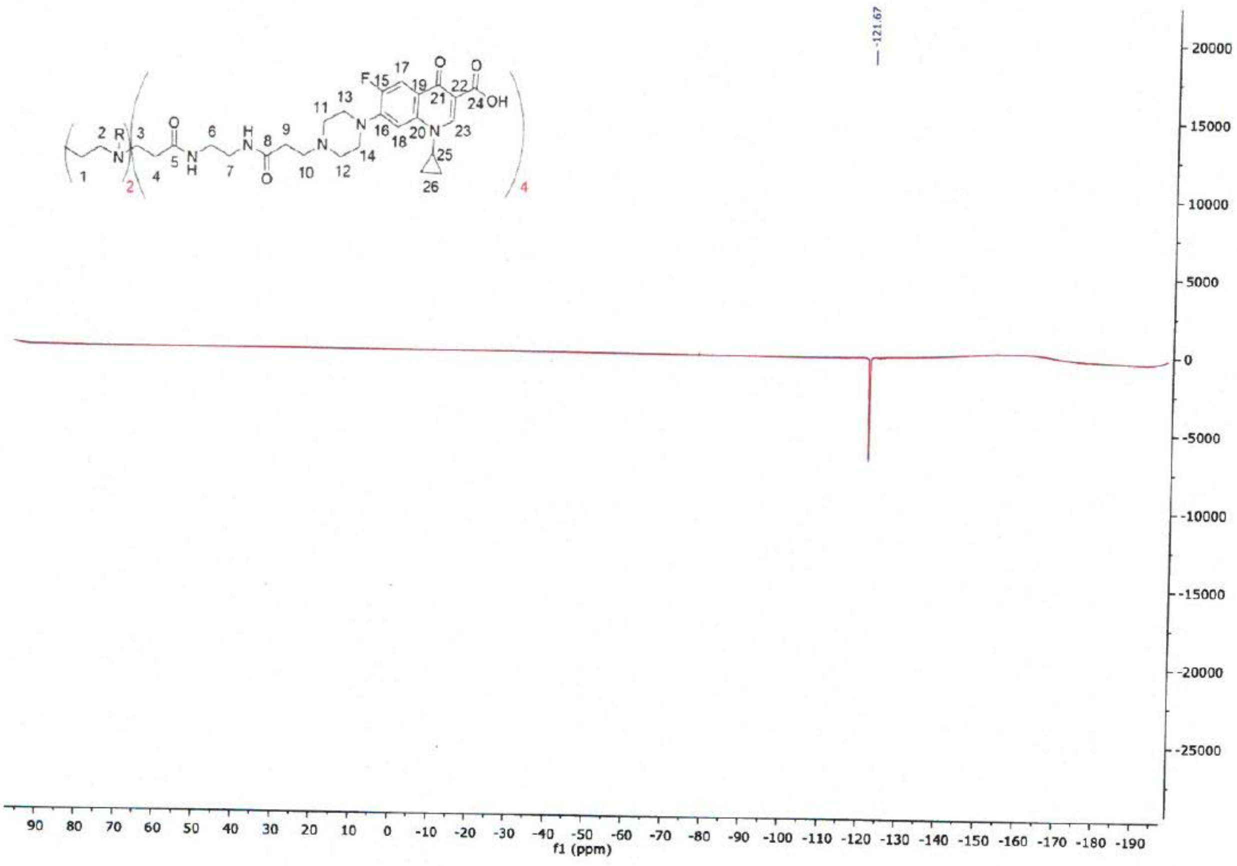

Figure S20: ESI-MS of **DAB-PAMAM-G0-(Cipro-COOH)$_4$ ● 6HCl (4).**

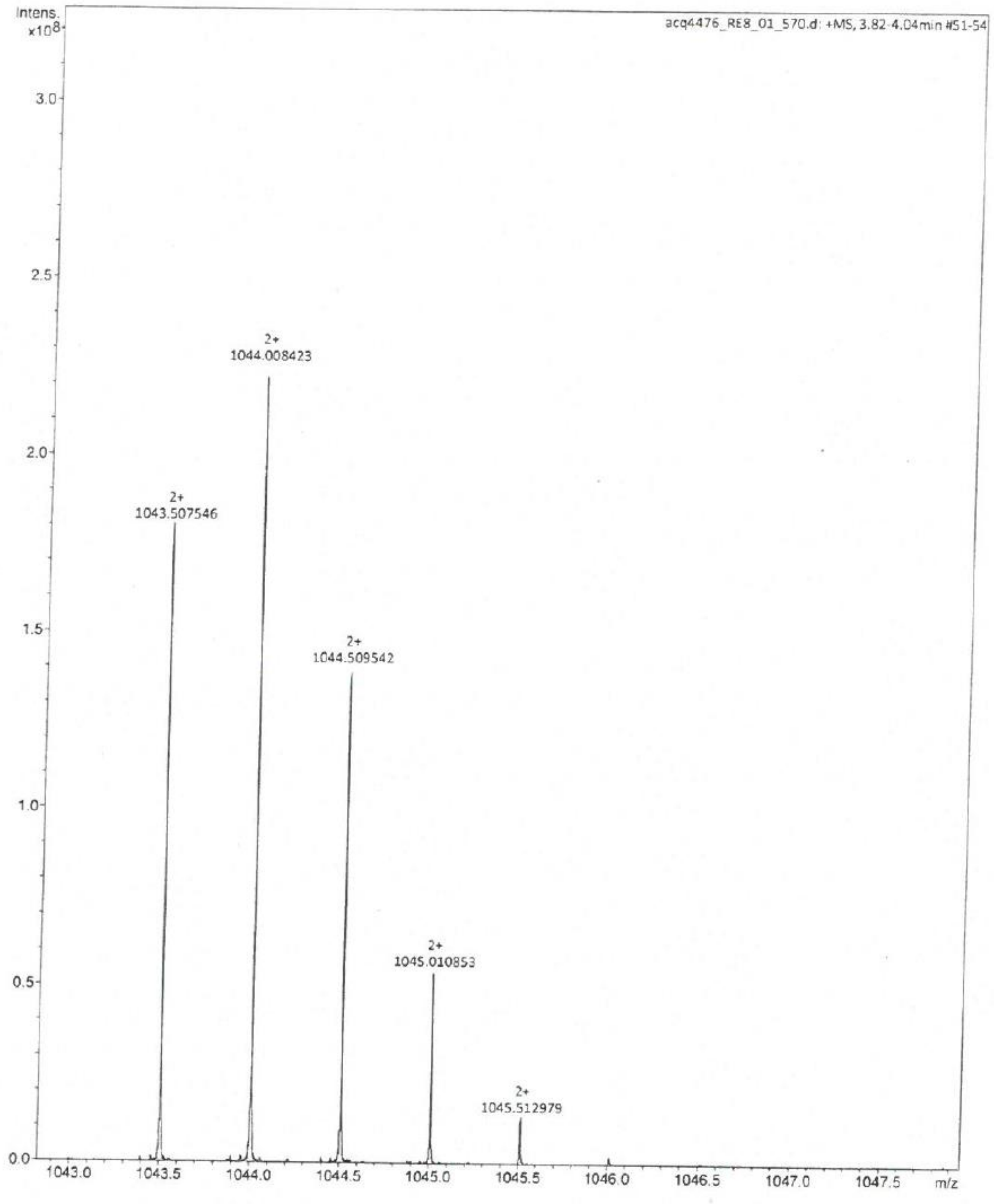

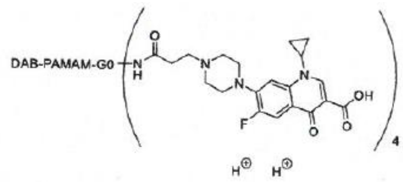

Chemical Formula: $C_{104}H_{134}F_4N_{22}O_{20}^{2+}$
Molecular Weight: 2088.3425
m/z: 1044.0052 (100.0%), 1043.5035 (88.9%), 1044.5069 (55.7%), 1045.0085 (10.4%), 1045.0085 (10.1%), 1044.5037 (8.1%), 1044.0020 (7.2%), 1045.5102 (4.6%), 1045.0073 (4.1%), 1044.5056 (3.7%), 1045.0054 (3.1%), 1045.5090 (2.3%), 1044.5083 (1.5%), 1045.5070 (1.5%), 1045.0054 (1.4%), 1044.0066 (1.4%), 1046.0119 (1.1%), 1045.5102 (1.0%)

Figure S21: ESI-MS of **DAB-PAMAM-G0-(Cipro-COOH)$_4$ • 6HCl (4).**

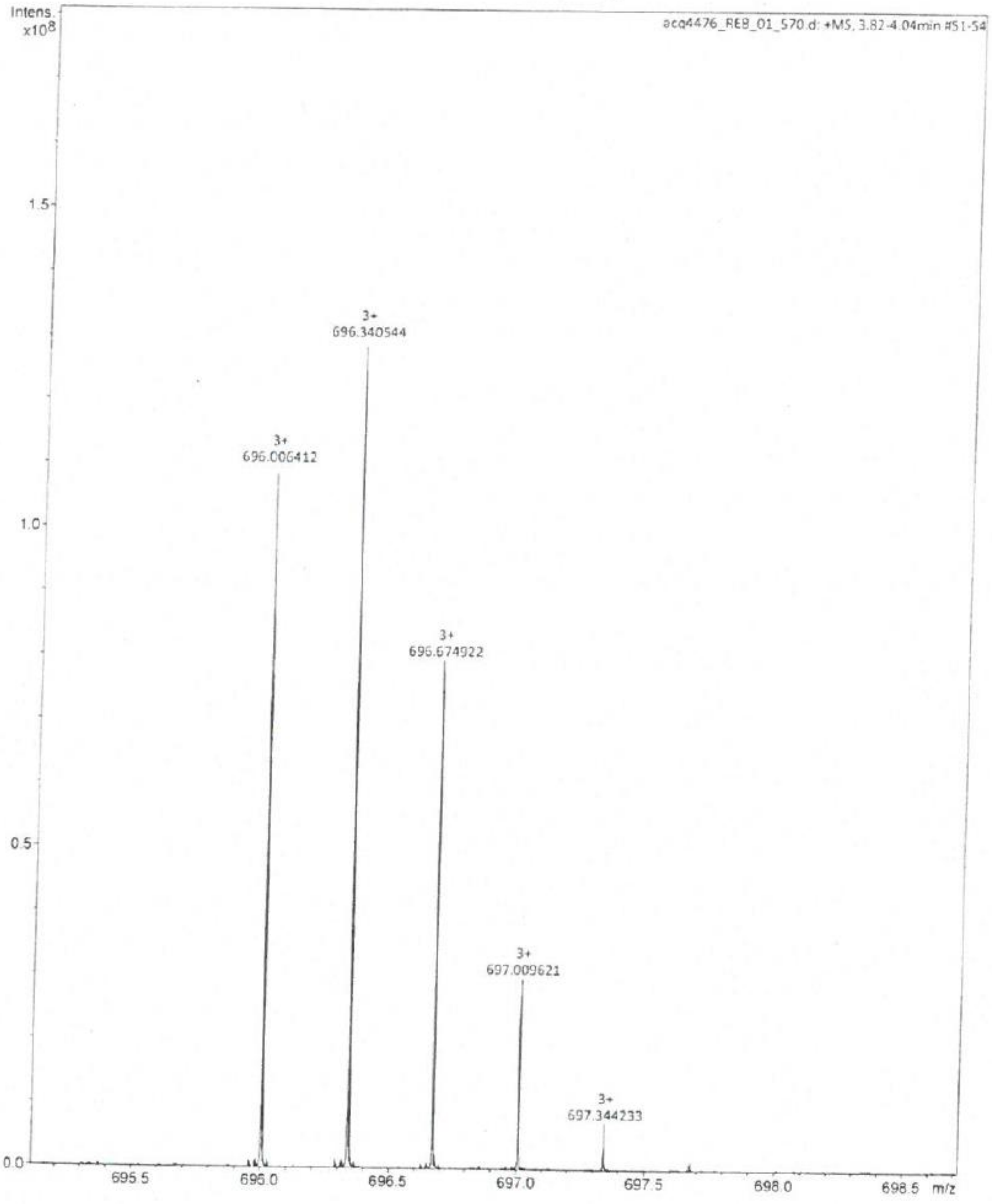

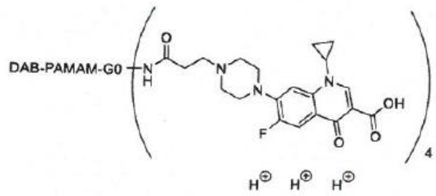

Chemical Formula: $C_{104}H_{135}F_4N_{22}O_{20}{}^{3+}$
Molecular Weight: 2089.3500
m/z: 696.3392 (100.0%), 696.0048 (88.9%), 696.6737 (38.0%), 697.0081 (18.9%), 696.6737 (17.7%), 696.6716 (8.1%), 696.3371 (7.2%), 697.3426 (5.5%), 697.0060 (4.5%), 697.0073 (4.1%), 696.6728 (3.7%), 697.0081 (1.6%), 697.3417 (1.6%), 696.6746 (1.6%), 696.3402 (1.4%), 697.6770 (1.2%)

Figure S22: ESI-MS of **DAB-PAMAM-G0-(Cipro-COOH)$_4$ • 6HCl (4).**

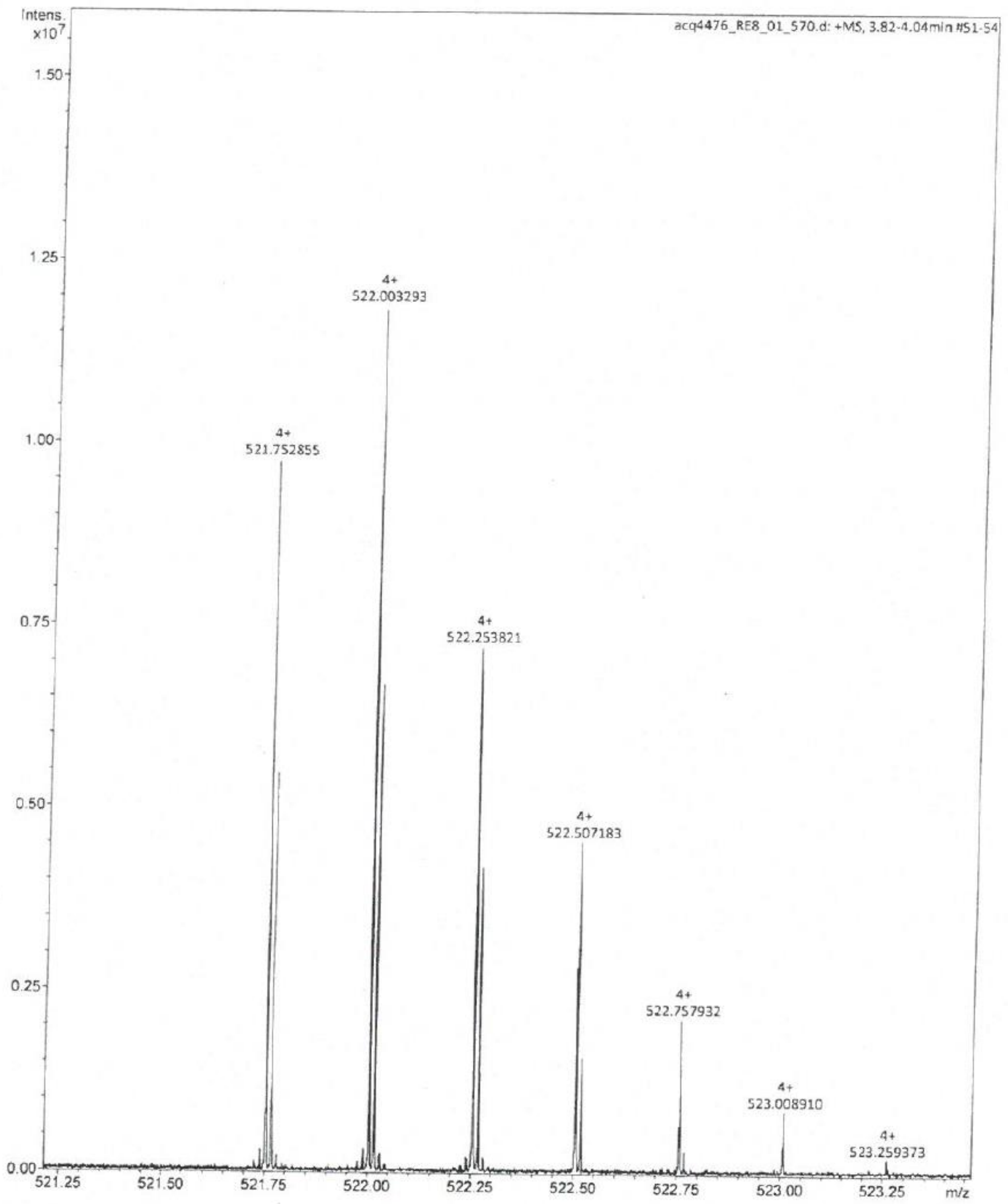

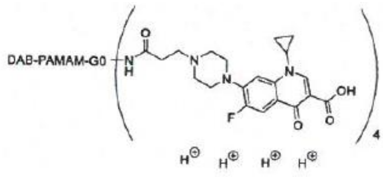

Chemical Formula: $C_{104}H_{136}F_4N_{22}O_{20}{}^{4+}$
Molecular Weight: 2090.3574
m/z: 522.5062 (100.0%), 522.2554 (88.9%), 522.7571 (55.7%), 523.0079 (10.4%), 523.0079 (10.1%), 522.7555 (8.1%), 522.5046 (7.2%), 523.2587 (4.6%), 523.0073 (4.1%), 522.7564 (3.7%), 523.0063 (3.1%), 523.2581 (2.3%), 522.7578 (1.6%), 523.2572 (1.5%), 523.0063 (1.4%), 522.5070 (1.4%), 523.5096 (1.1%), 523.2587 (1.0%)

Figure S23: ^{1}H-NMR (500 MHz in D_2O) of **DAB-PAMAM-G0-(Acetamide)$_4$ ● 2HCl (5)**

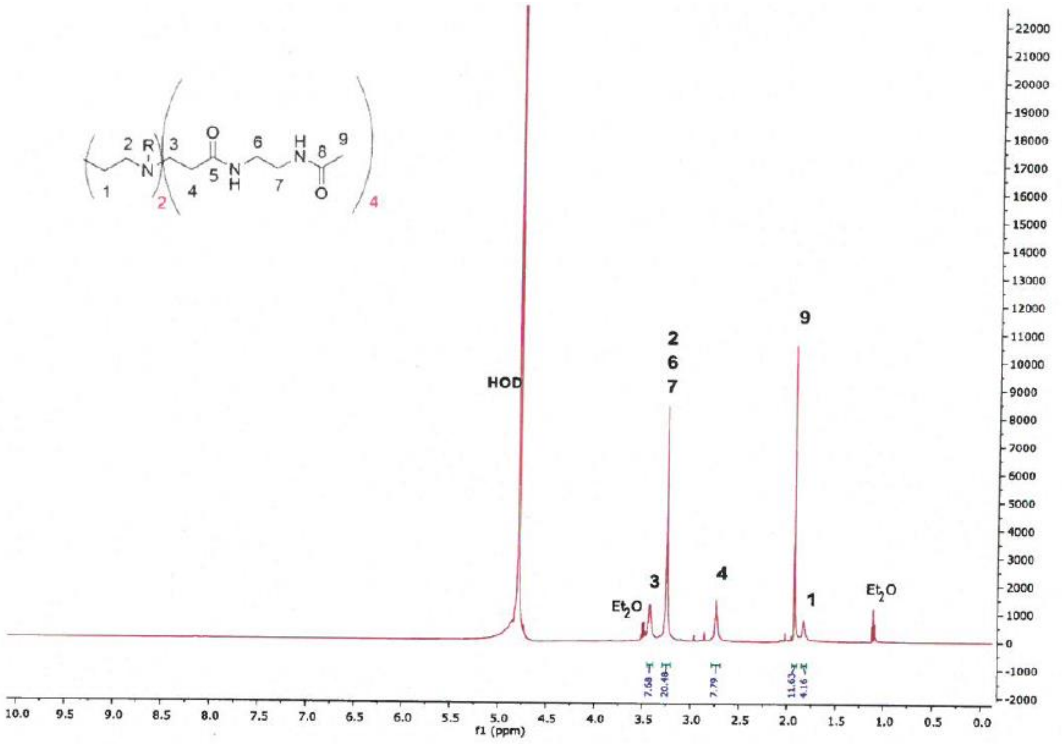

Figure S24: ^{13}C-NMR (125 MHz in D_2O) of **DAB-PAMAM-G0-(Acetamide)$_4$ ● 2HCl (5)**

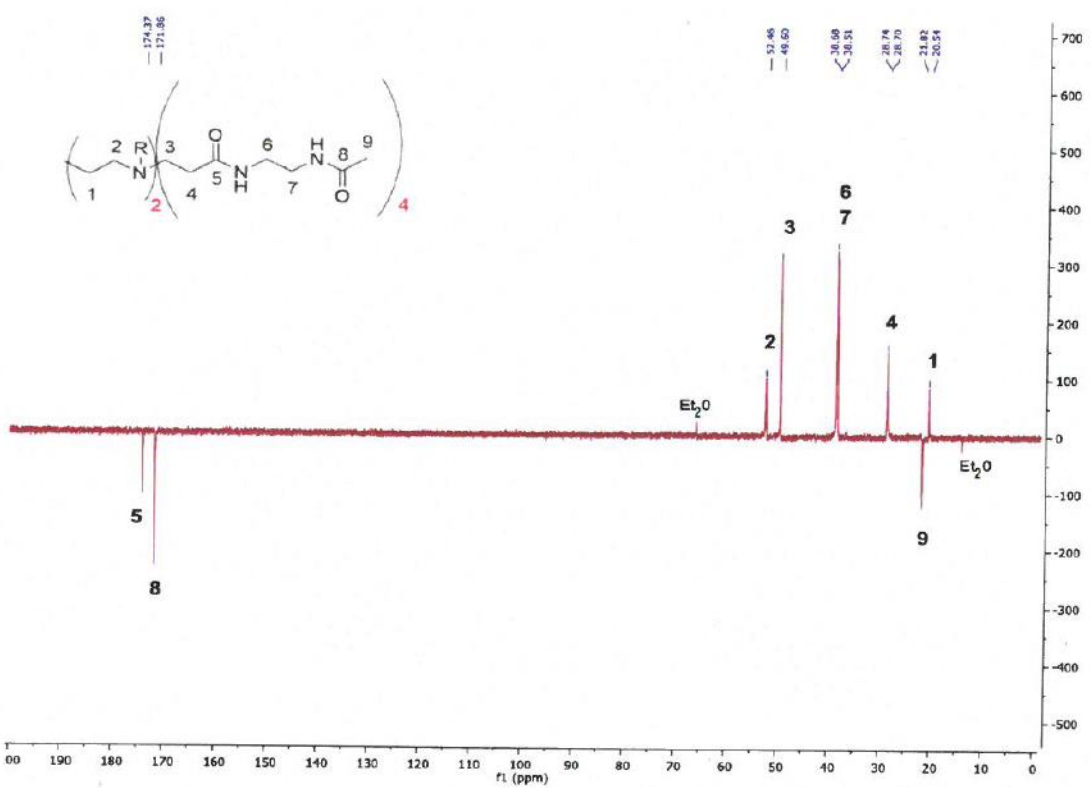

Figure S25: COSY-NMR (500 MHz in D_2O) of **DAB-PAMAM-G0-(Acetamide)$_4$ • 2HCl (5)**

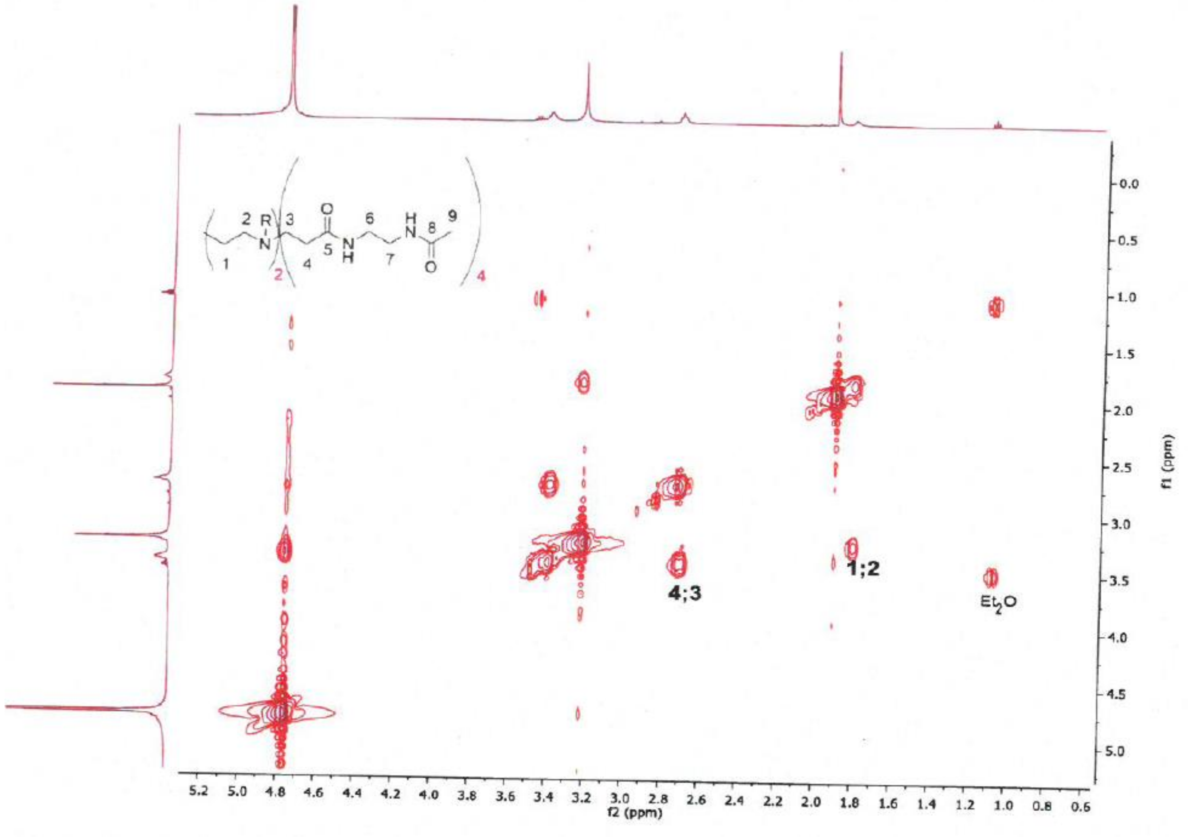

Figure S26: HSQC-NMR (125 & 500 MHz in D_2O) of **DAB-PAMAM-G0-(Acetamide)$_4$ • 2HCl (5)**

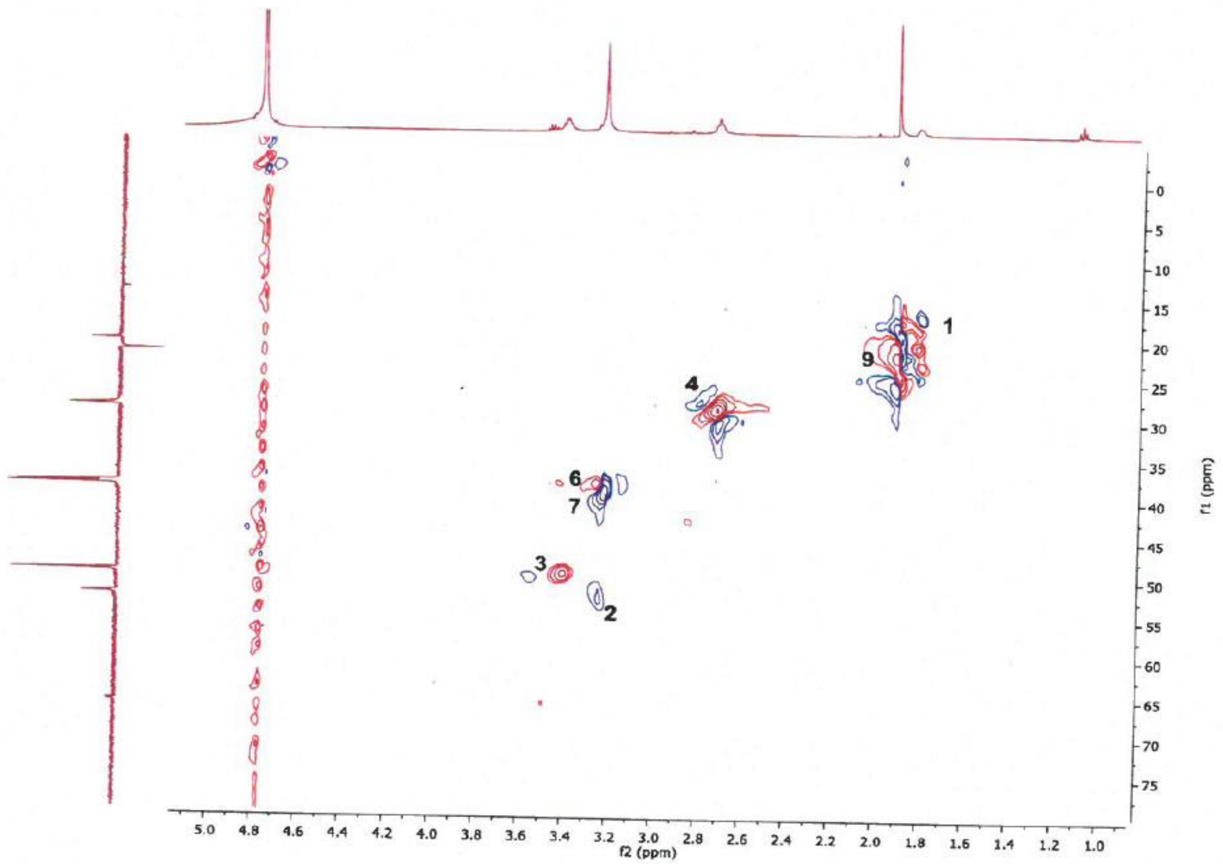

Figure S27: ESI-MS of DAB-PAMAM-G0-(Acetamide)$_4$ ● 2HCl (5)

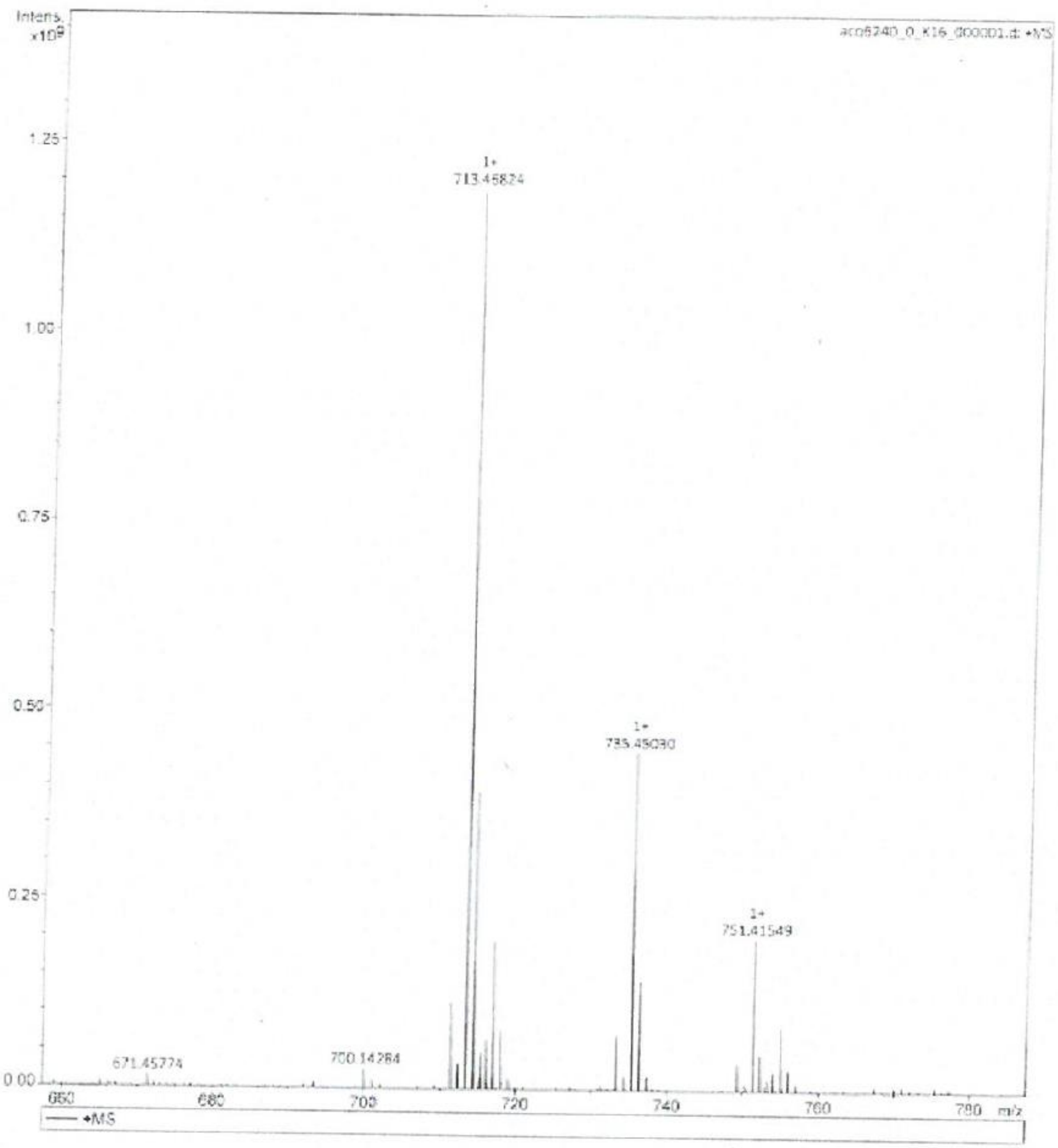

Figure SP47: Mass analysis (MALDI)(Matrix:Dithranol). m/z $[M+H^+]^+$ calcd for $[C_{32}H_{61}N_{10}O_6]^+$: 713.4686; Found: 713.4682; m/z $[M+Na^+]^+$ calcd for $[C_{32}H_{60}N_{10}NaO_6]^+$: 735.4488; Found: 735.4503; m/z $[M+K^+]^+$ calcd for $[C_{32}H_{60}N_{10}KO_6]^+$: 751.4227; Found: 751.4155

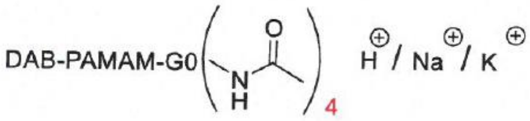

Figure S28: ESI-MS of DAB-PAMAM-G0-(Acetamide)$_4$ • 2HCl (5)

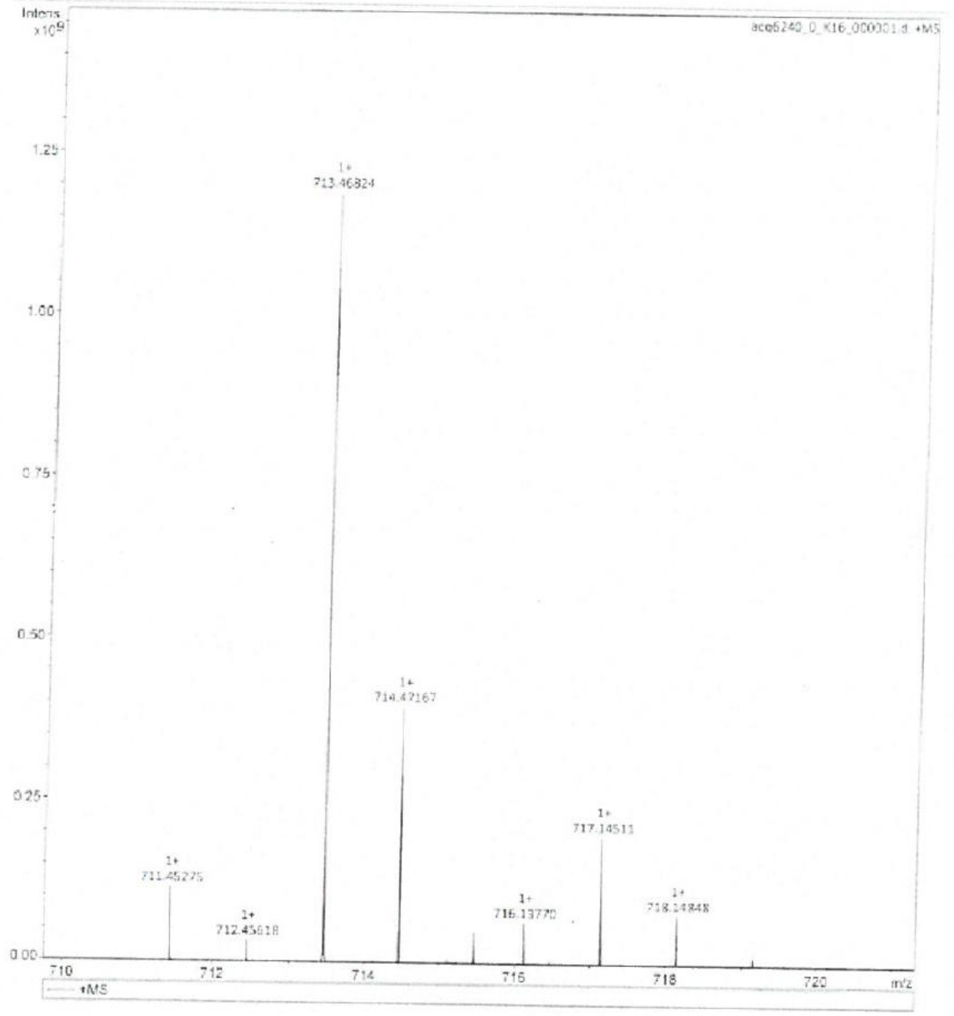

Figure SP48: Mass analysis (MALDI)(Matrix:Dithranol). m/z $[M+H^+]^+$ calcd for $[C_{32}H_{61}N_{10}O_6]^+$: 713.4686;
Found: 713.4682

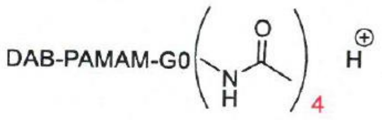

Chemical Formula: $C_{32}H_{61}N_{10}O_8^+$
Molecular Weight: 713.9015
m/z: 713.4668 (100.0%), 714.4702 (34.6%), 715.4735 (3.1%), 715.4735 (2.7%), 714.4639 (1.8%), 714.4639 (1.8%), 715.4711 (1.6%), 715.4672 (1.3%)

Figure S29: ^{1}H-NMR (500 MHz in $CDCl_3$) of Benzyl 7-(4-tert-butoxycarbonyl)piperazin-1-yl)-1-cyclopropyl-6-fluoro-4-oxo-1,4-dihydroquinoline-3-carboxylate (7)

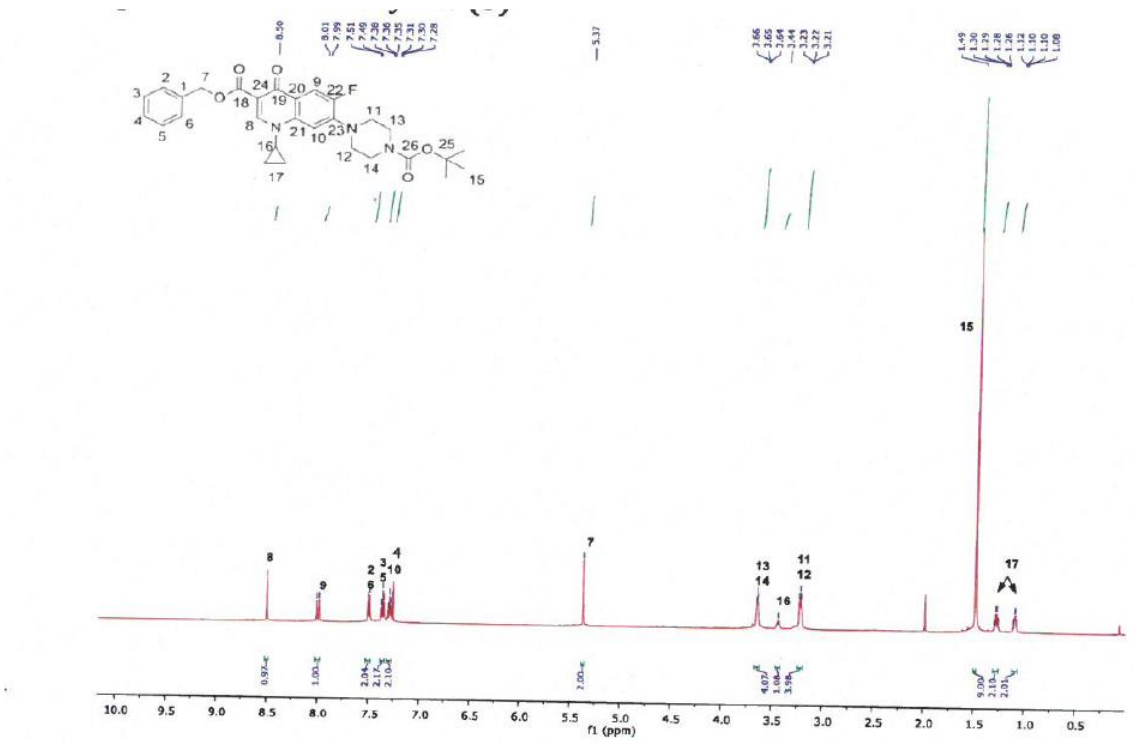

Figure S30: COSY-NMR (500 MHz in $CDCl_3$) of Benzyl 7-(4-tert-butoxycarbonyl)piperazin-1-yl)-1-cyclopropyl-6-fluoro-4-oxo-1,4-dihydroquinoline-3-carboxylate (7)

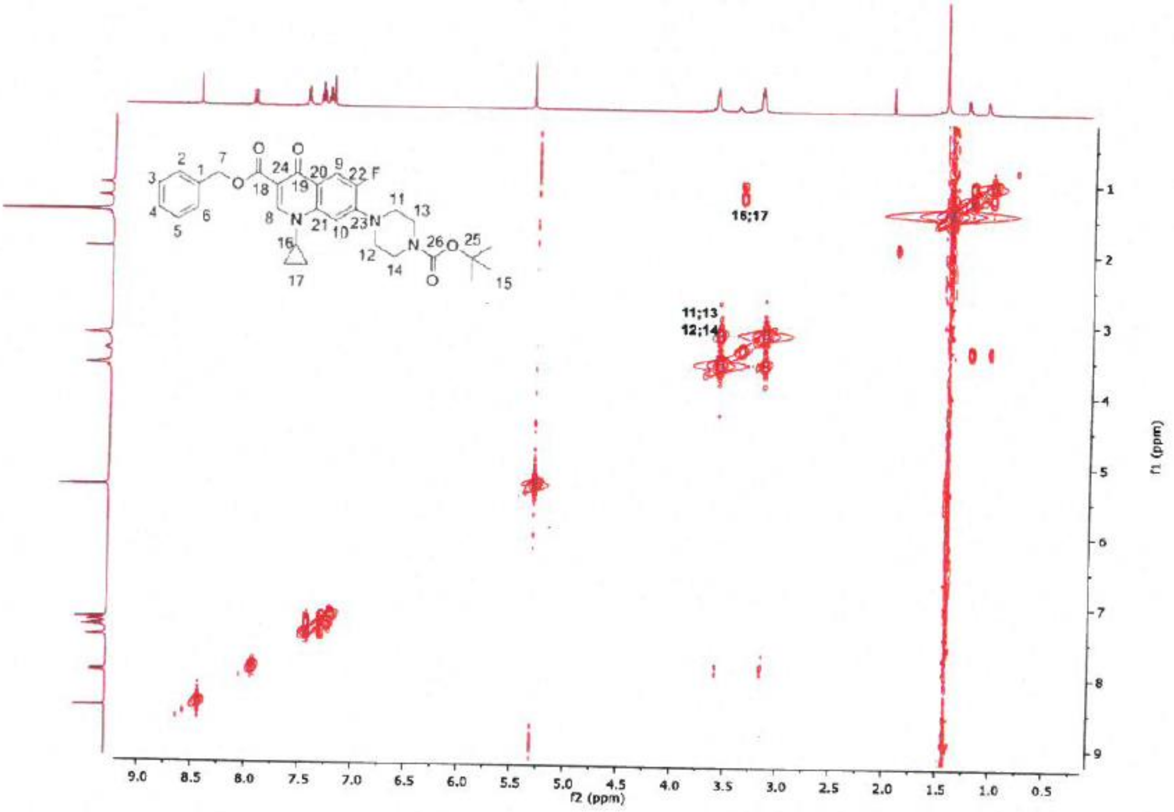

Figure S30: ^{13}C-NMR (125 MHz in $CDCl_3$) of Benzyl 7-(4-tert-butoxycarbonyl)piperazin-1-yl)-1-cyclopropyl-6-fluoro-4-oxo-1,4-dihydroquinoline-3-carboxylate (7)

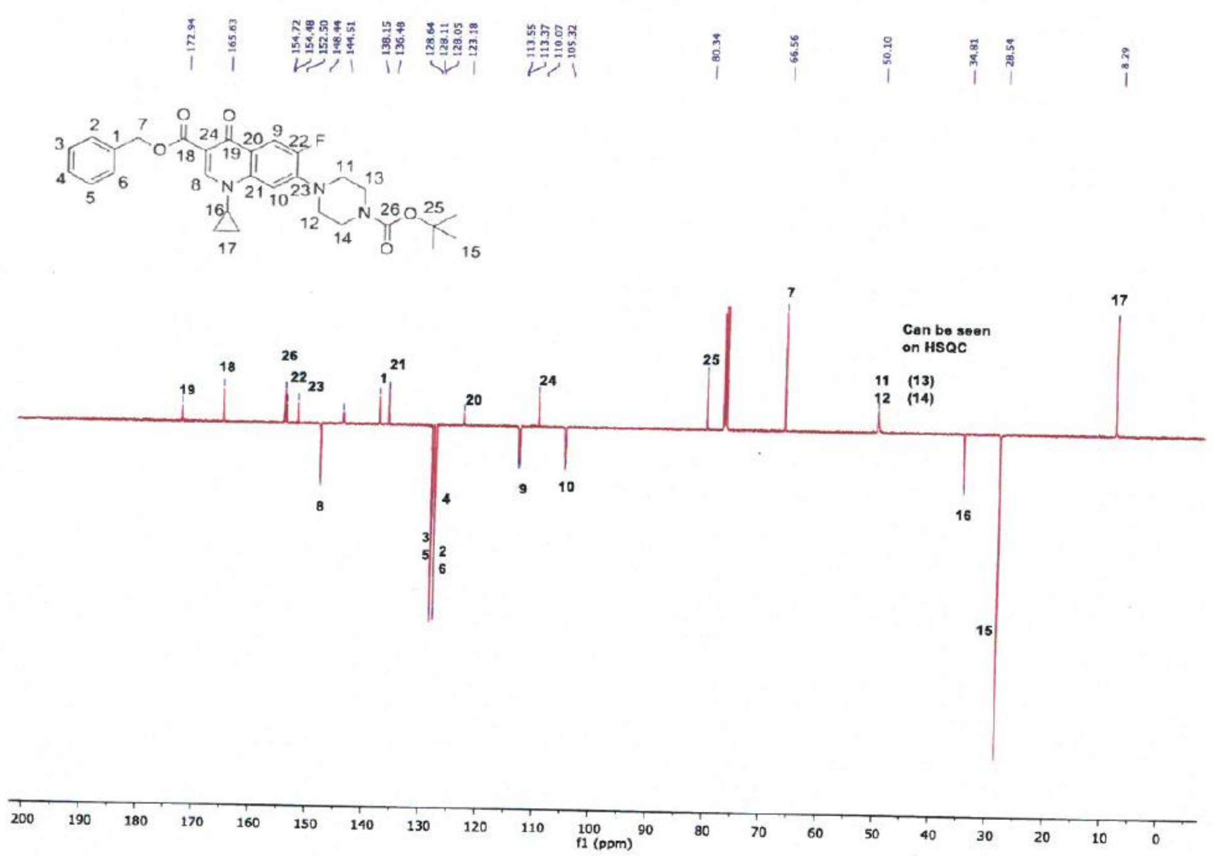

Figure S31: HSQC-NMR (125 & 500 MHz in CDCl3) of Benzyl 7-(4-tert-butoxycarbonyl)piperazin-1-yl)-1-cyclopropyl-6-fluoro-4-oxo-1,4-dihydroquinoline-3-carboxylate (7)

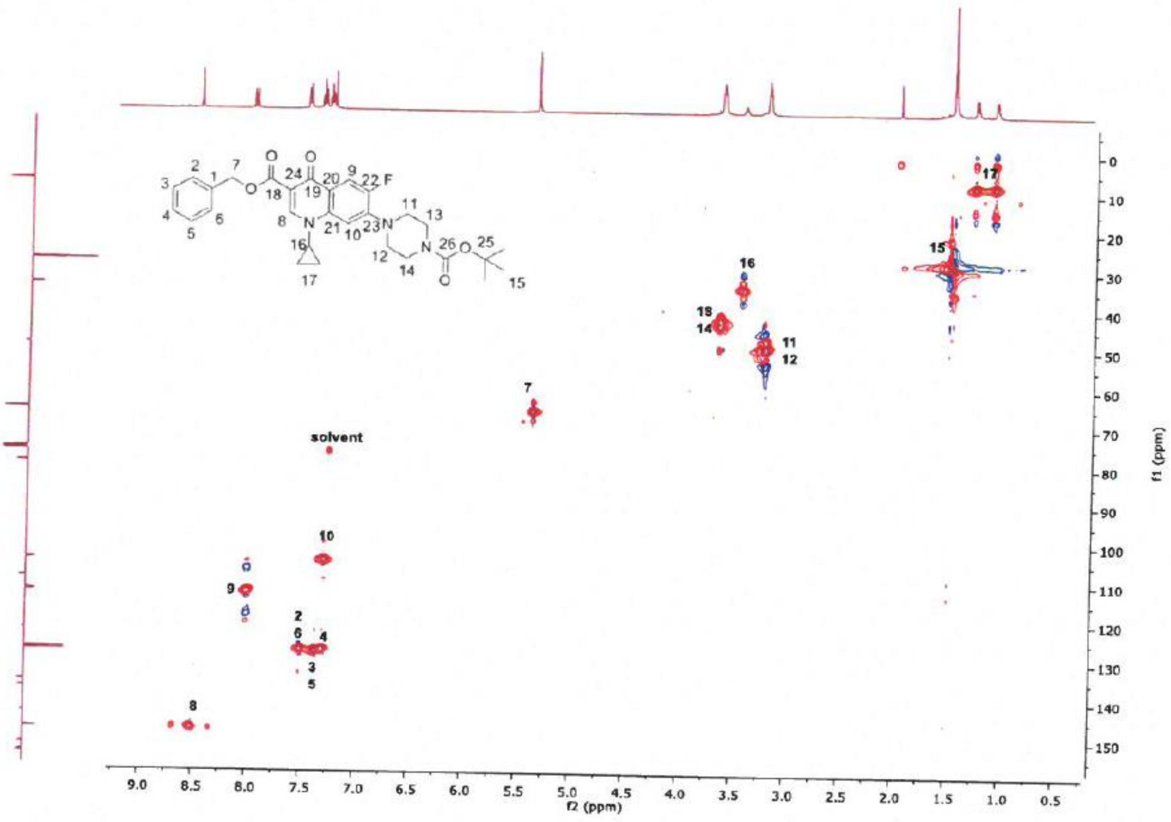

Figure S32: ^{19}F-NMR (470 MHz in $CDCl_3$) of Benzyl 7-(4-tert-butoxycarbonyl)piperazin-1-yl)-1-cyclopropyl-6-fluoro-4-oxo-1,4-dihydroquinoline-3-carboxylate (7)

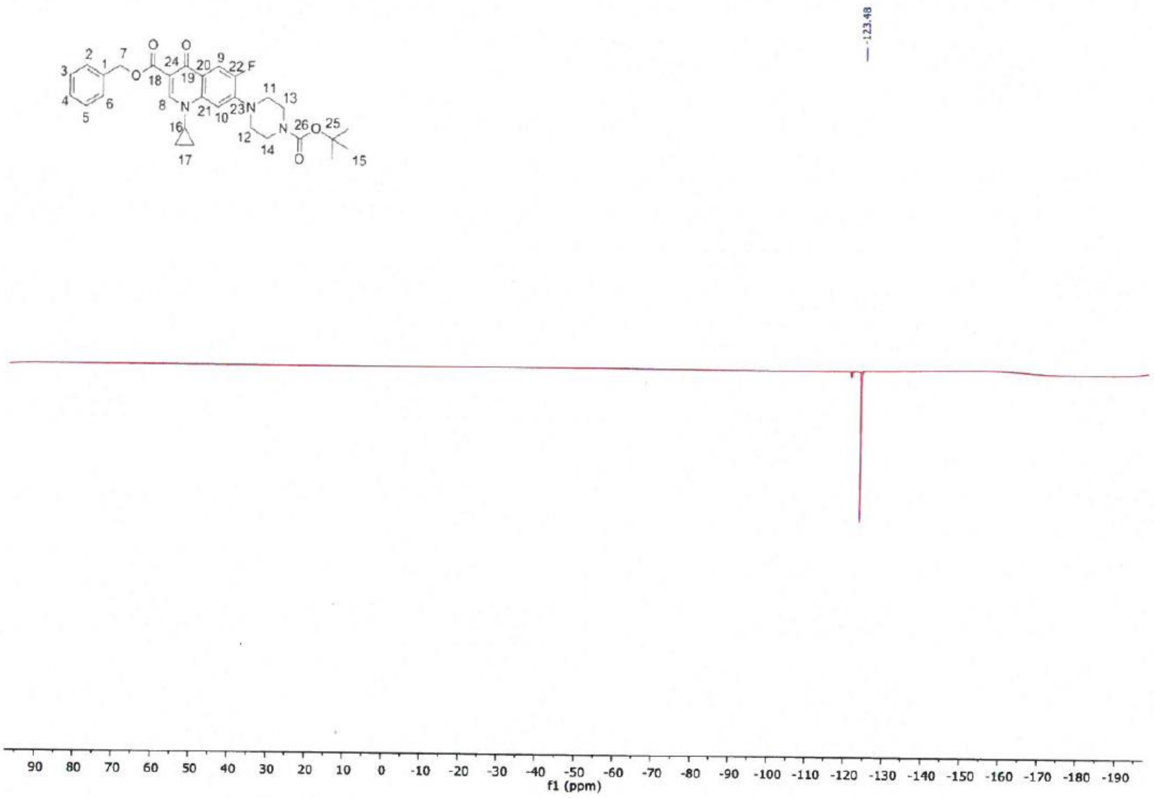

Figure S33: ESI-MS of Benzyl 7-(4-tert-butoxycarbonyl)piperazin-1-yl)-1-cyclopropyl-6-fluoro-4-oxo-1,4-dihydroquinoline-3-carboxylate (7)

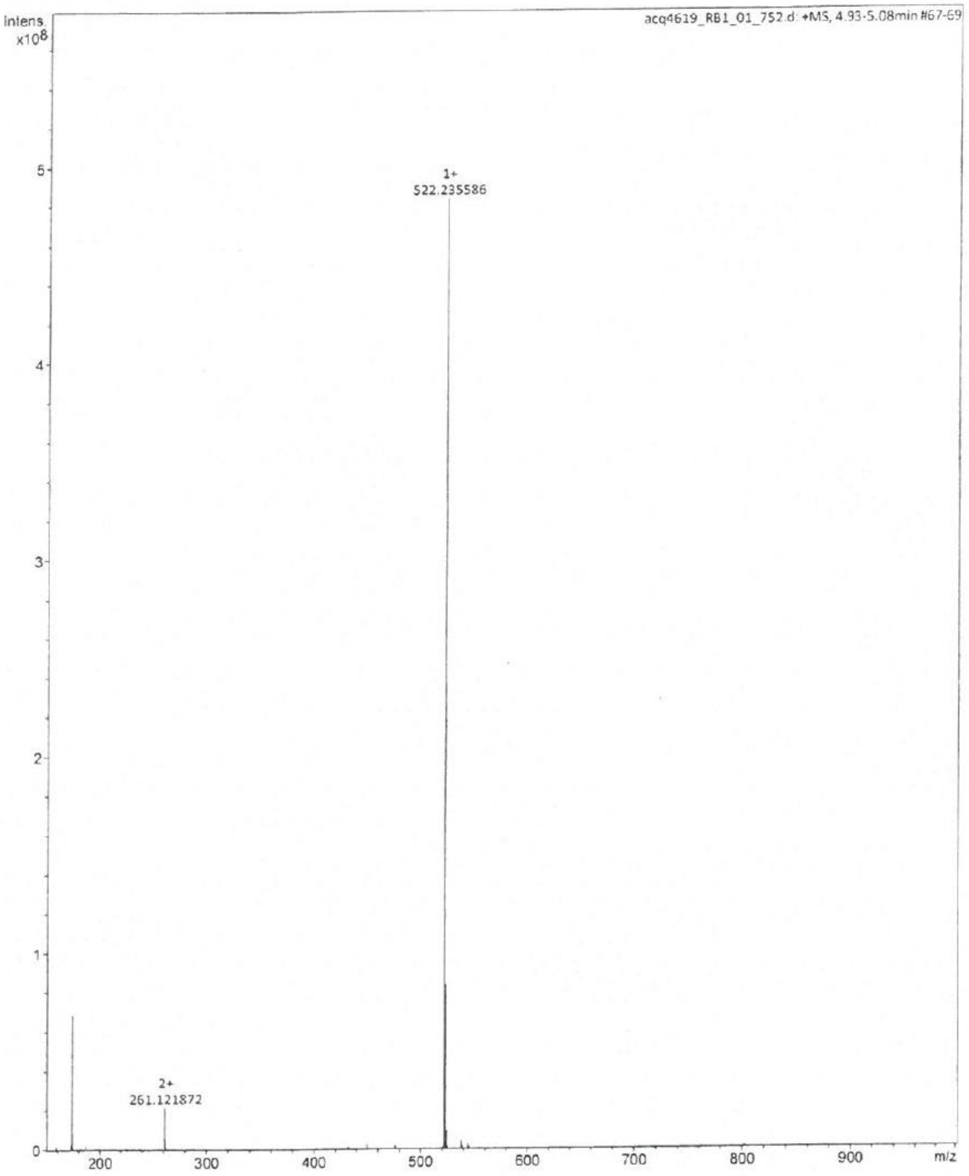

Figure SP10: Mass analysis (ESI)(MeOH+0.1%TFA). m/z $[M+2H^{+}]^{2+}$ calcd for $[C_{29}H_{34}FN_3O_5]^{2+}$: 261.6236; Found: 261.1219

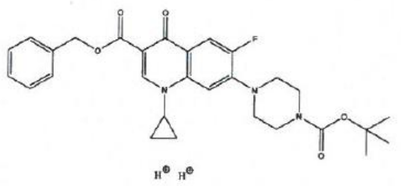

Chemical Formula: $C_{29}H_{34}FN_3O_5^{2+}$
Molecular Weight: 523.6043
m/z: 261.6236 (100.0%), 262.1253 (31.4%), 262.6269 (4.7%), 262.1221 (1.1%), 262.6257 (1.0%)

Figure S34: ESI-MS of Benzyl 7-(4-tert-butoxycarbonyl)piperazin-1-yl)-1-cyclopropyl-6-fluoro-4-oxo-1,4-dihydroquinoline-3-carboxylate (7)

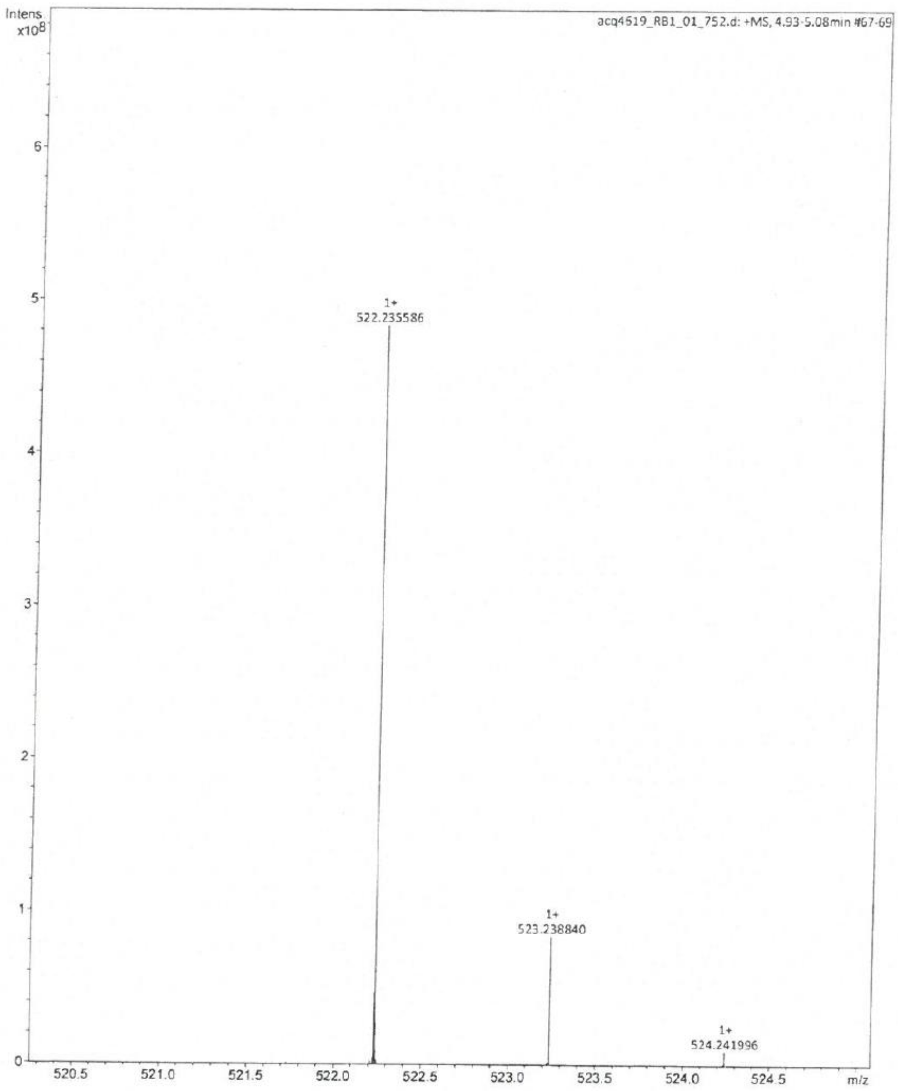

Figure SP11: Mass analysis (ESI)(MeOH+0.1%TFA). m/z $[M+H^+]^+$ calcd for $[C_{29}H_{33}FN_3O_5]^+$: 522.2399; Found: 522.2356

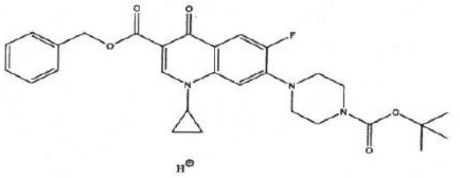

Chemical Formula: $C_{29}H_{33}FN_3O_5^+$

Molecular Weight: 522.5969

m/z: 522.2399 (100.0%), 523.2432 (31.4%), 524.2466 (2.7%), 524.2466 (2.0%), 523.2369 (1.1%), 524.2441 (1.0%)

Figure S35: ^{1}H-NMR (500 MHz in $CDCl_3$) of **Benzyl 1-cyclopropyl-6-fluoro-4-oxo-piperazin-1-yl)-1,4-dihydroquinoline-3-carboxylate (8)**

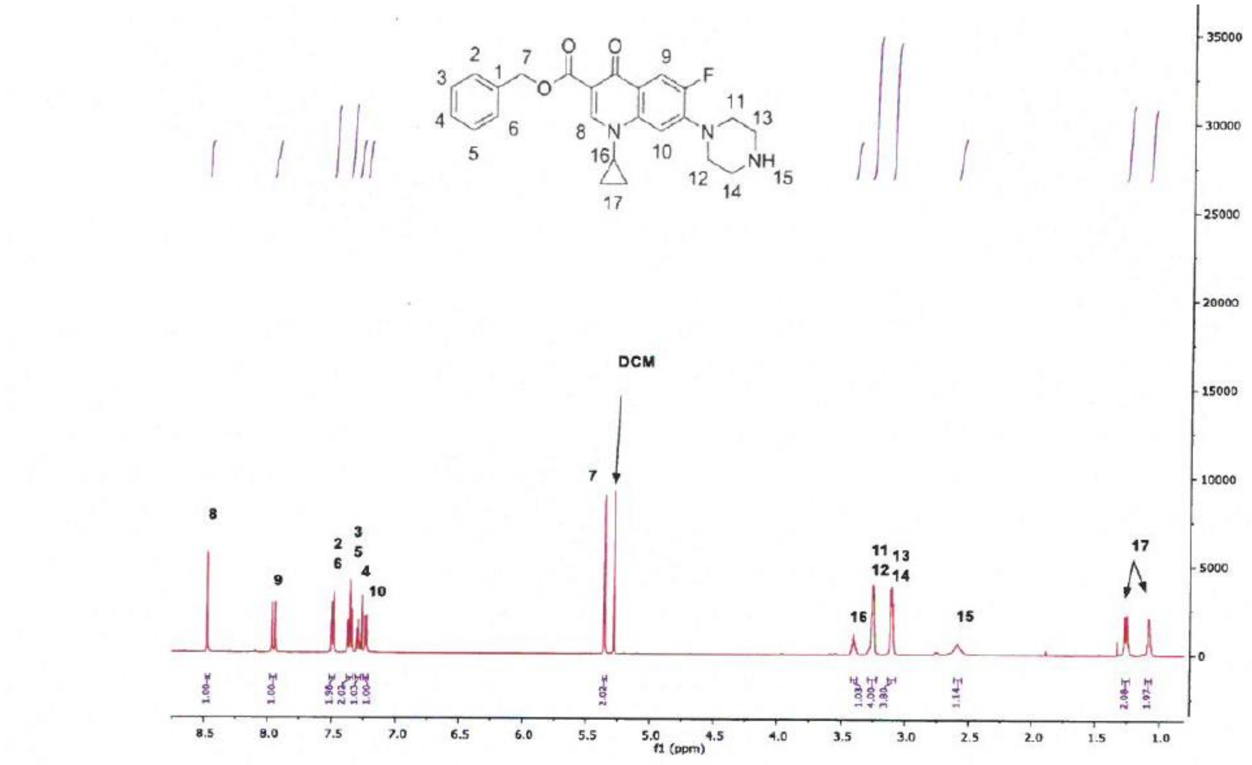

Figure S36: COSY-NMR (500 MHz in $CDCl_3$) of **Benzyl 1-cyclopropyl-6-fluoro-4-oxo-piperazin-1-yl)-1,4-dihydroquinoline-3-carboxylate (8)**

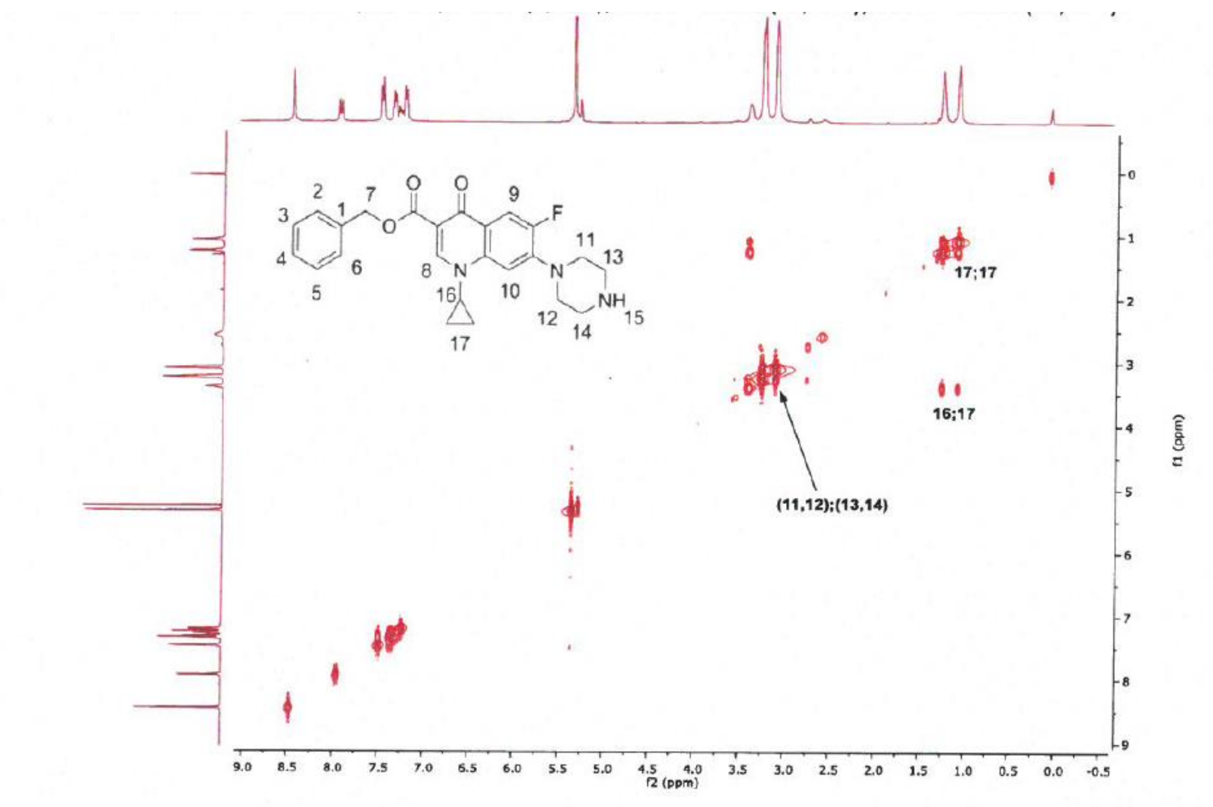

Figure S37: ^{13}C-NMR (125 MHz in $CDCl_3$) of **Benzyl 1-cyclopropyl-6-fluoro-4-oxo-piperazin-1-yl)-1,4-dihydroquinoline-3-carboxylate (8)**

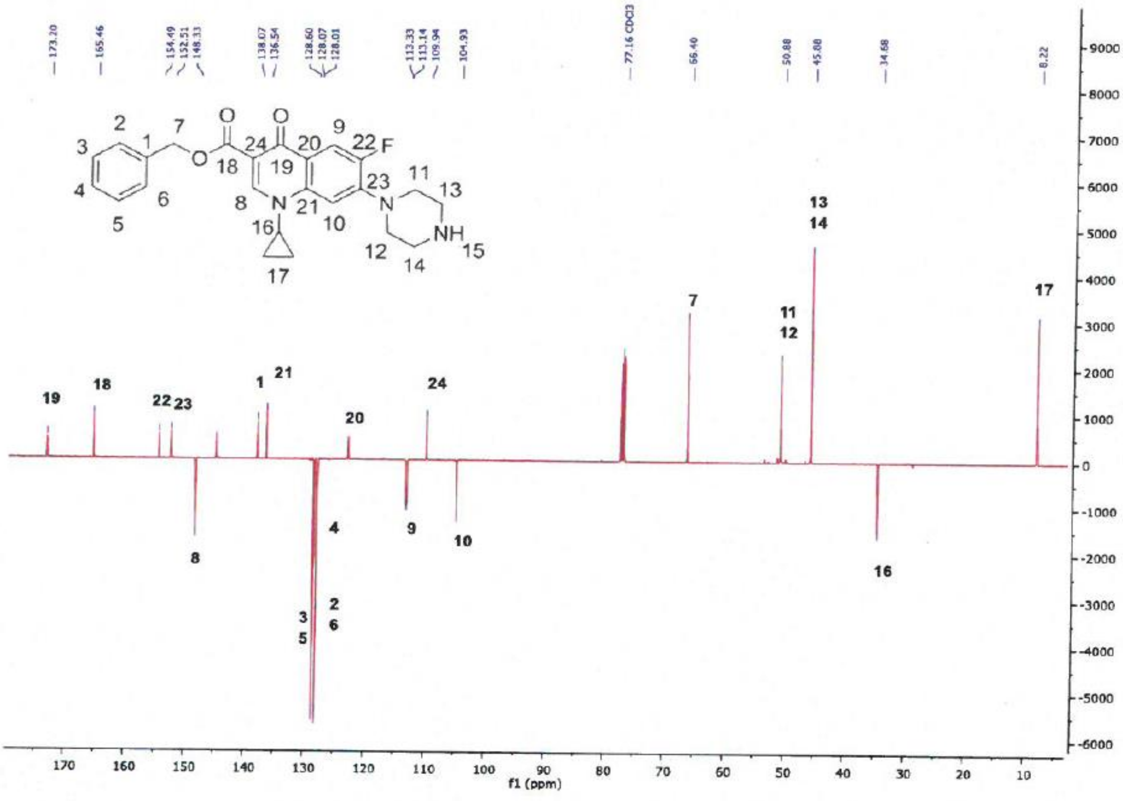

Figure S38: HSQC-NMR (125 & 500 MHz in $CDCl_3$) of **Benzyl 1-cyclopropyl-6-fluoro-4-oxo-piperazin-1-yl)-1,4-dihydroquinoline-3-carboxylate (8)**

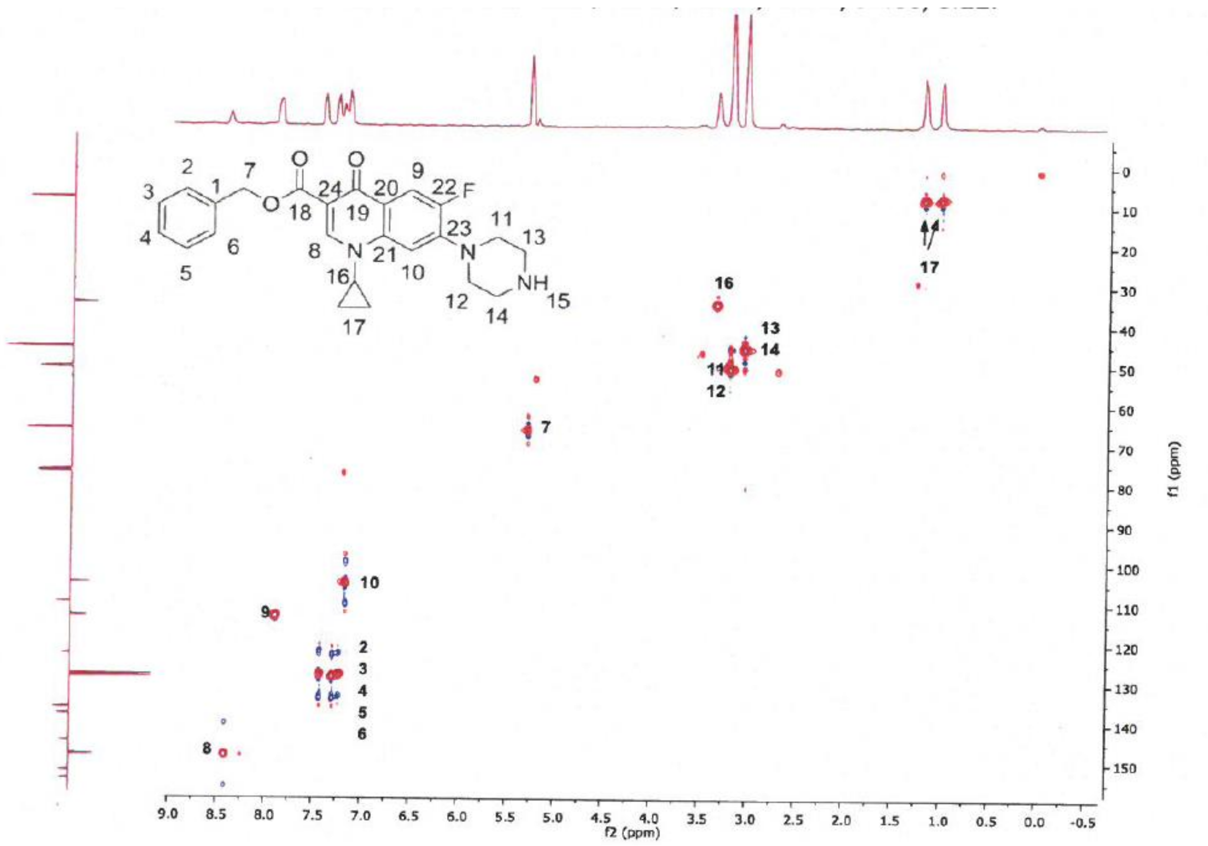

Figure S39: ^{19}F-NMR (470 MHz in $CDCl_3$) of **Benzyl 1-cyclopropyl-6-fluoro-4-oxo-piperazin-1-yl)-1,4-dihydroquinoline-3-carboxylate (8)**

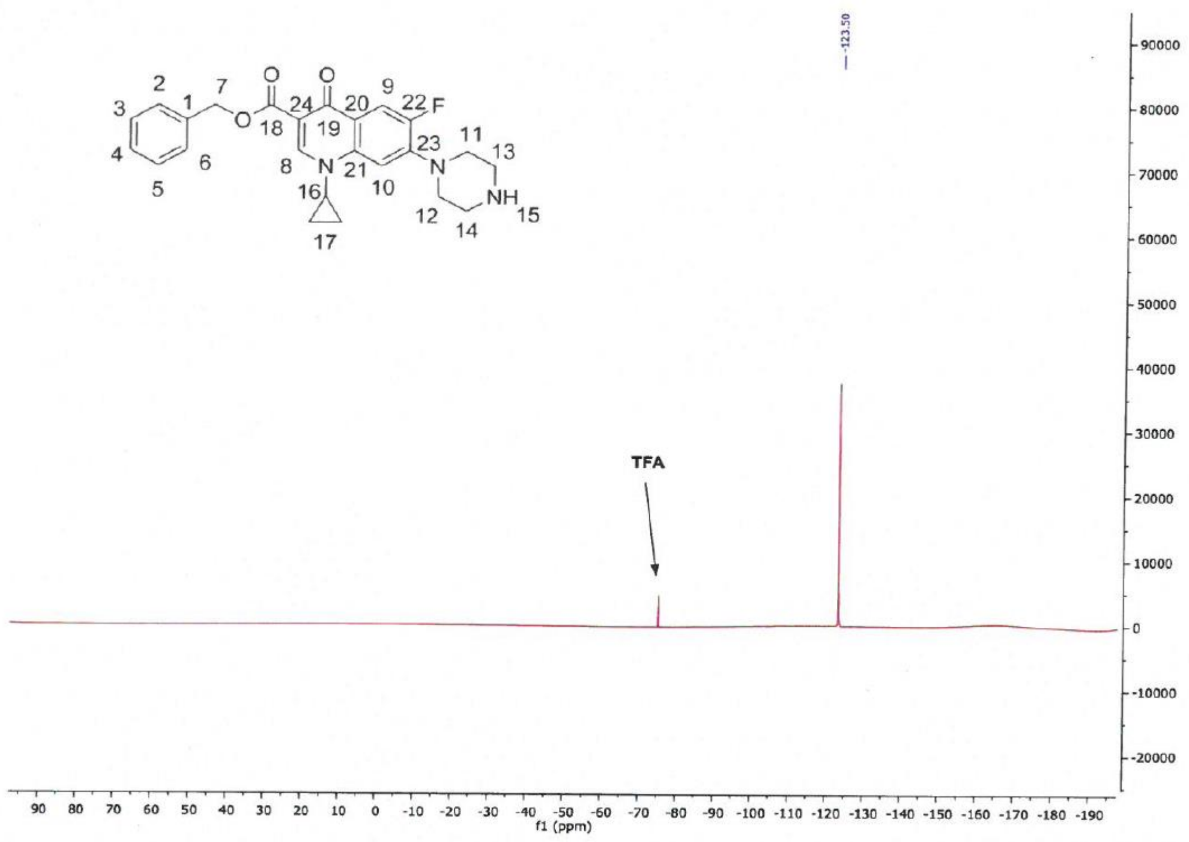

Figure S40: ESI-MS of **Benzyl 1-cyclopropyl-6-fluoro-4-oxo-piperazin-1-yl)-1,4-dihydroquinoline-3-carboxylate (8)**

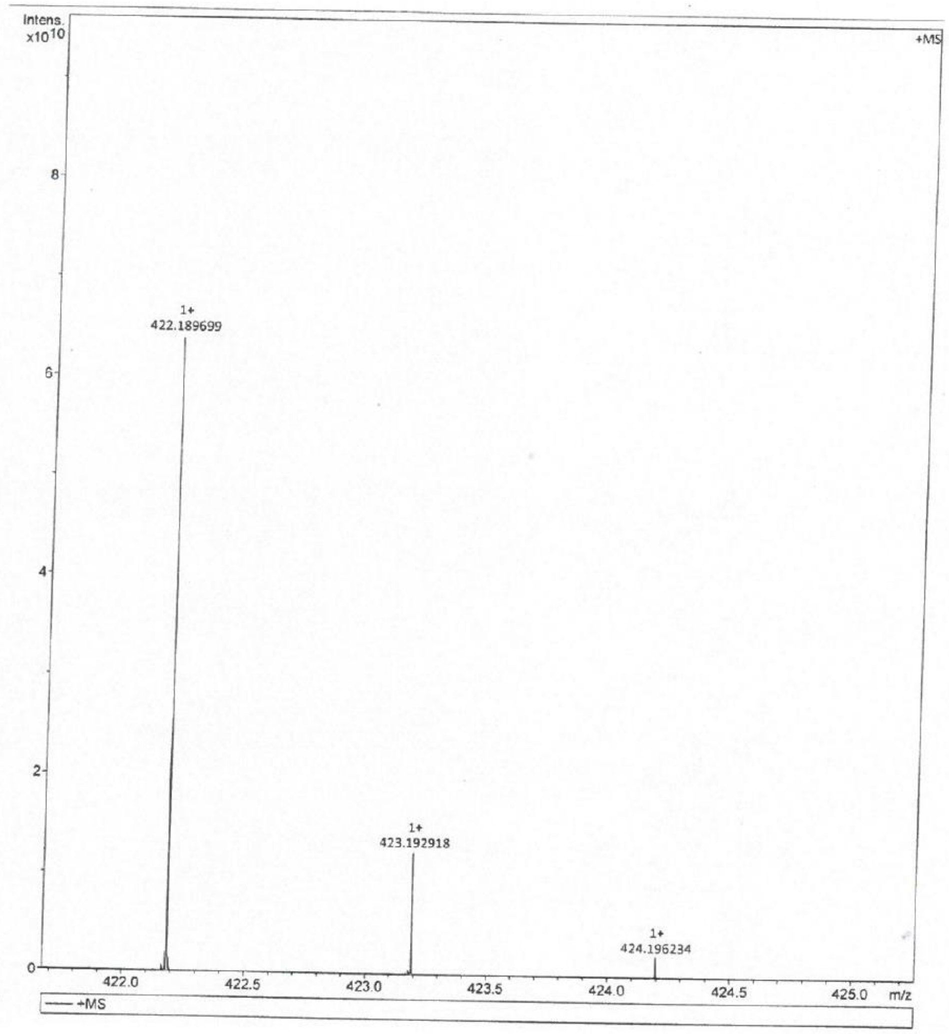

Figure SP17: Mass analysis (ESI)(MeOH+0.1%TFA). m/z $[M+H^+]^+$ calcd for $[C_{24}H_{25}FN_3O_3]^+$: 422.1875; Found: 422.1897

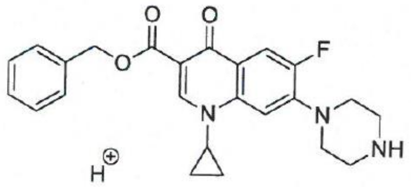

Chemical Formula: $C_{24}H_{25}FN_3O_3^+$

Molecular Weight: 422.4715

m/z: 422.1875 (100.0%), 423.1908 (26.0%), 424.1942 (3.2%), 423.1845 (1.1%)

Figure S41: ^{1}H-NMR (500 MHz in $CDCl_3$) of **Benzyl 1-cyclopropyl-6-fluoro-4-oxo-7-(4-(3-oxo-3-phenoxypropyl)piperazin-1-yl)- 1,4-dihydroquinoline-3-carboxylate (9)**

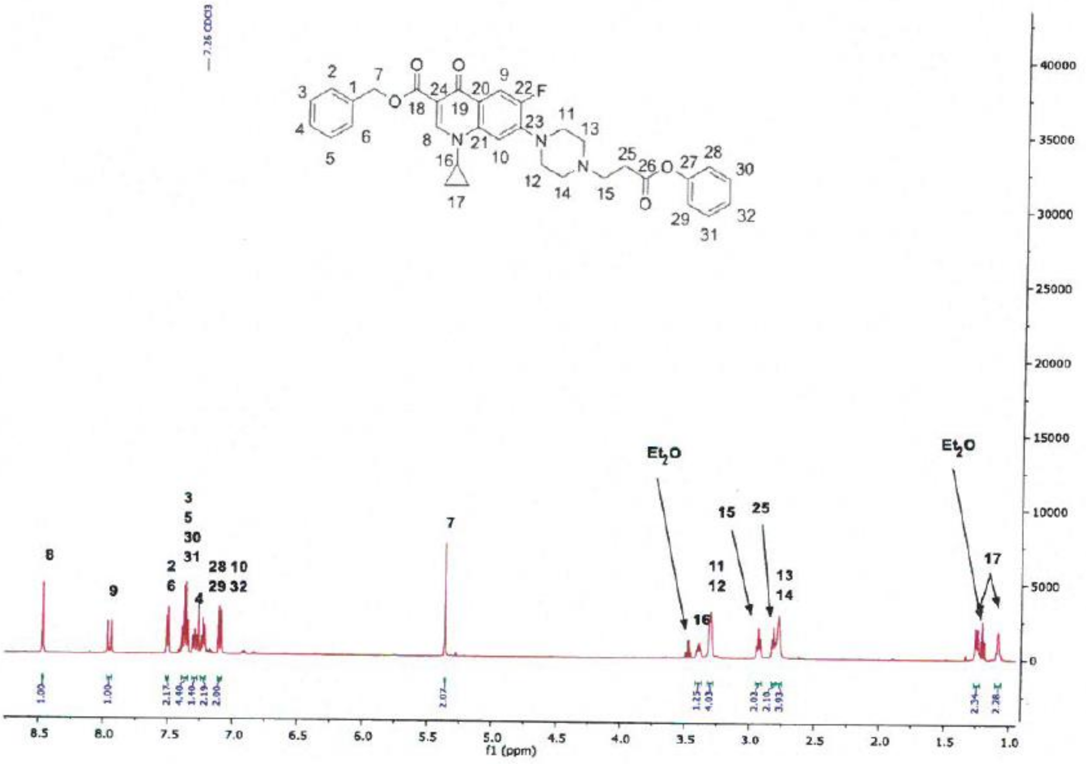

Figure S42: COSY-NMR (500 MHz in $CDCl_3$) of **Benzyl 1-cyclopropyl-6-fluoro-4-oxo-7-(4-(3-oxo-3-phenoxypropyl)piperazin-1-yl)- 1,4-dihydroquinoline-3-carboxylate (9)**

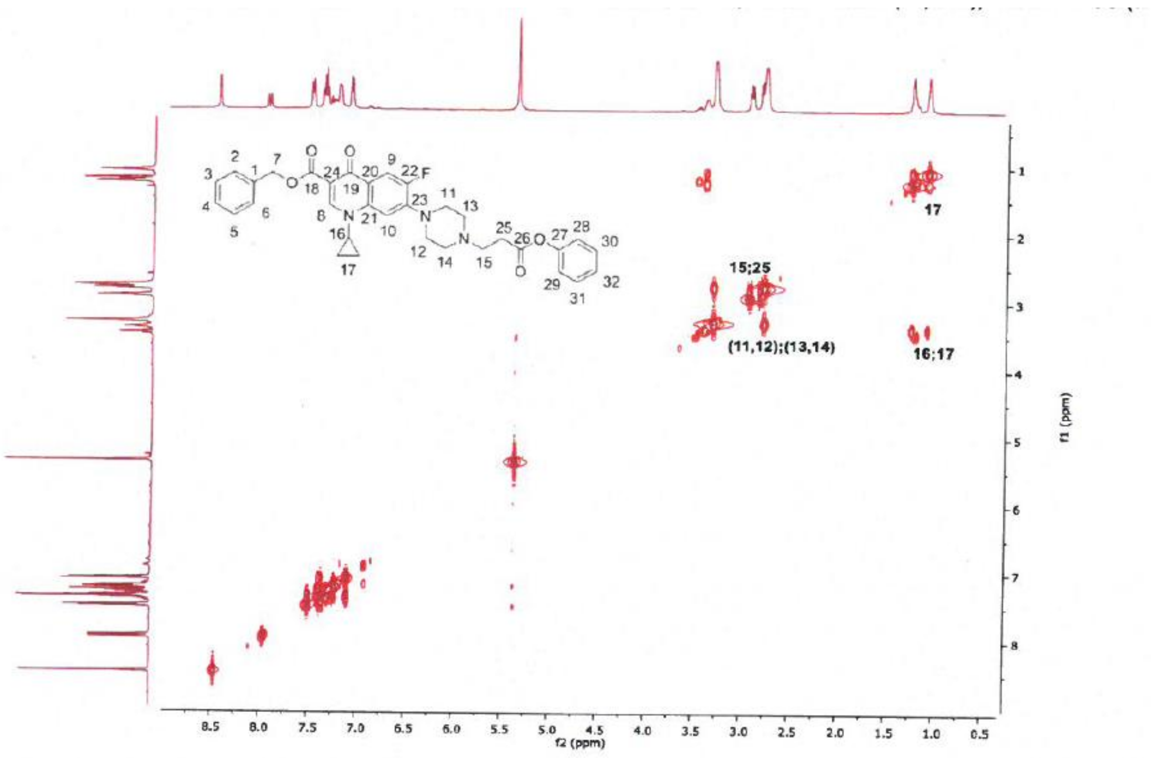

Figure S43: ^{13}C-NMR (125 MHz in $CDCl_3$) of **Benzyl 1-cyclopropyl-6-fluoro-4-oxo-7-(4-(3-oxo-3-phenoxypropyl)piperazin-1-yl)- 1,4-dihydroquinoline-3-carboxylate (9)**

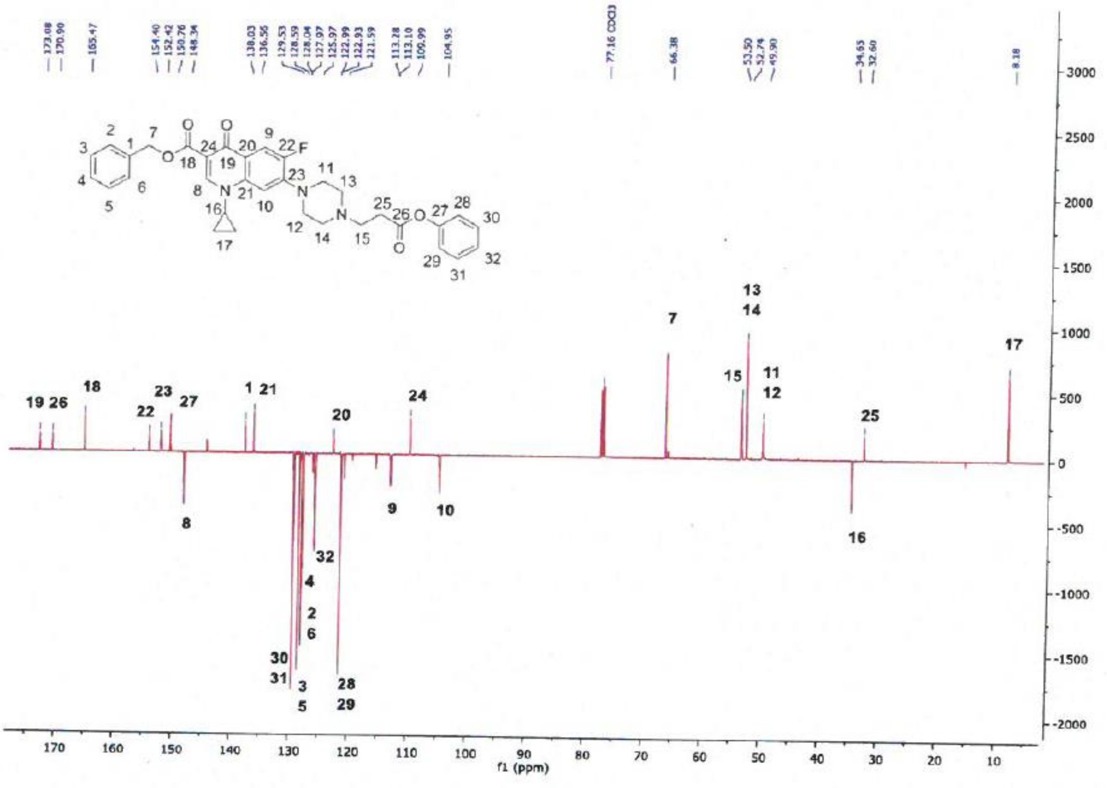

Figure S44: HSQC-NMR (125 & 500 MHz in $CDCl_3$) of **Benzyl 1-cyclopropyl-6-fluoro-4-oxo-7-(4-(3-oxo-3-phenoxypropyl)piperazin-1-yl)- 1,4-dihydroquinoline-3-carboxylate (9)**

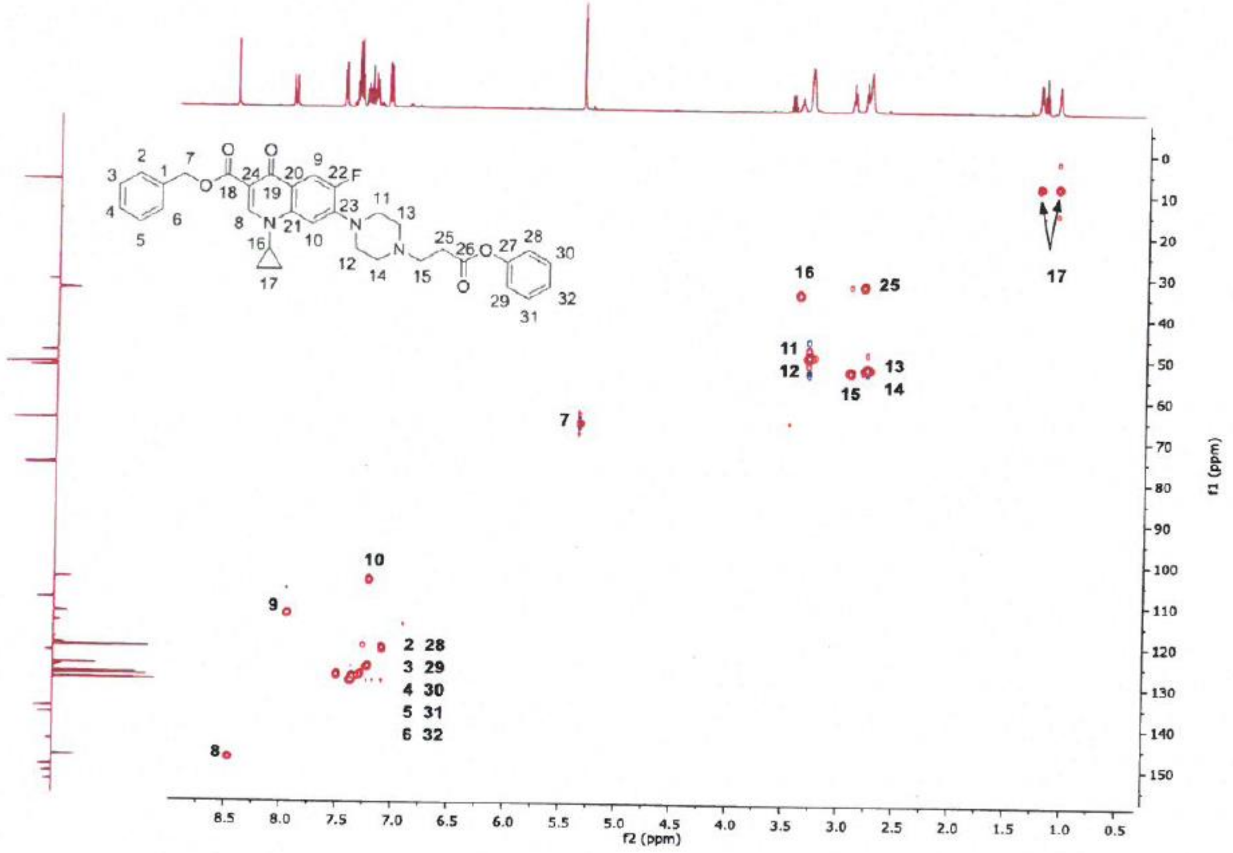

Figure S45: ^{19}F-NMR (470 MHz in $CDCl_3$) of **Benzyl 1-cyclopropyl-6-fluoro-4-oxo-7-(4-(3-oxo-3-phenoxypropyl)piperazin-1-yl)- 1,4-dihydroquinoline-3-carboxylate (9)**

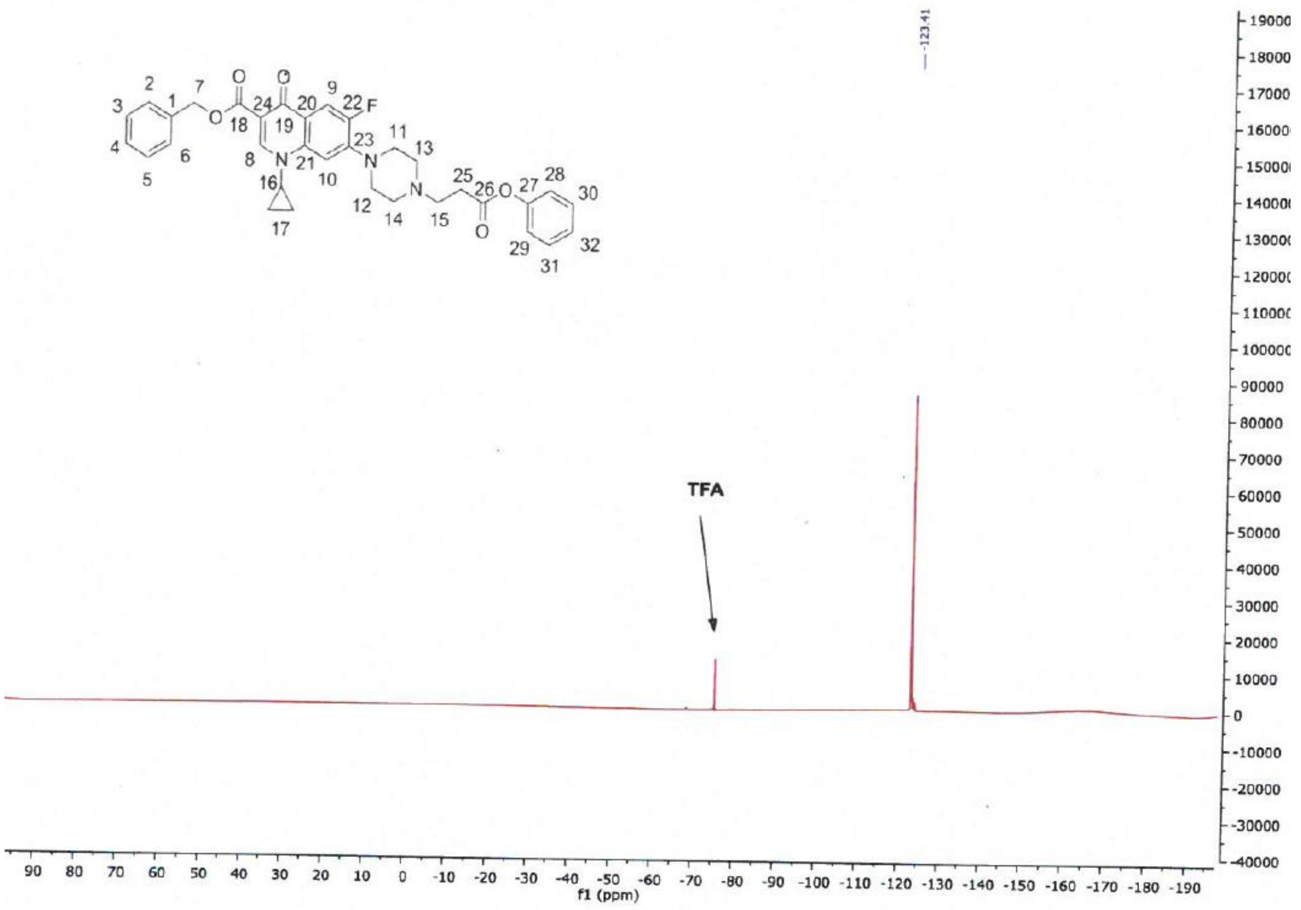

Figure S46: ESI-MS of **Benzyl 1-cyclopropyl-6-fluoro-4-oxo-7-(4-(3-oxo-3-phenoxypropyl)piperazin-1-yl)-1,4-dihydroquinoline-3-carboxylate (9)**

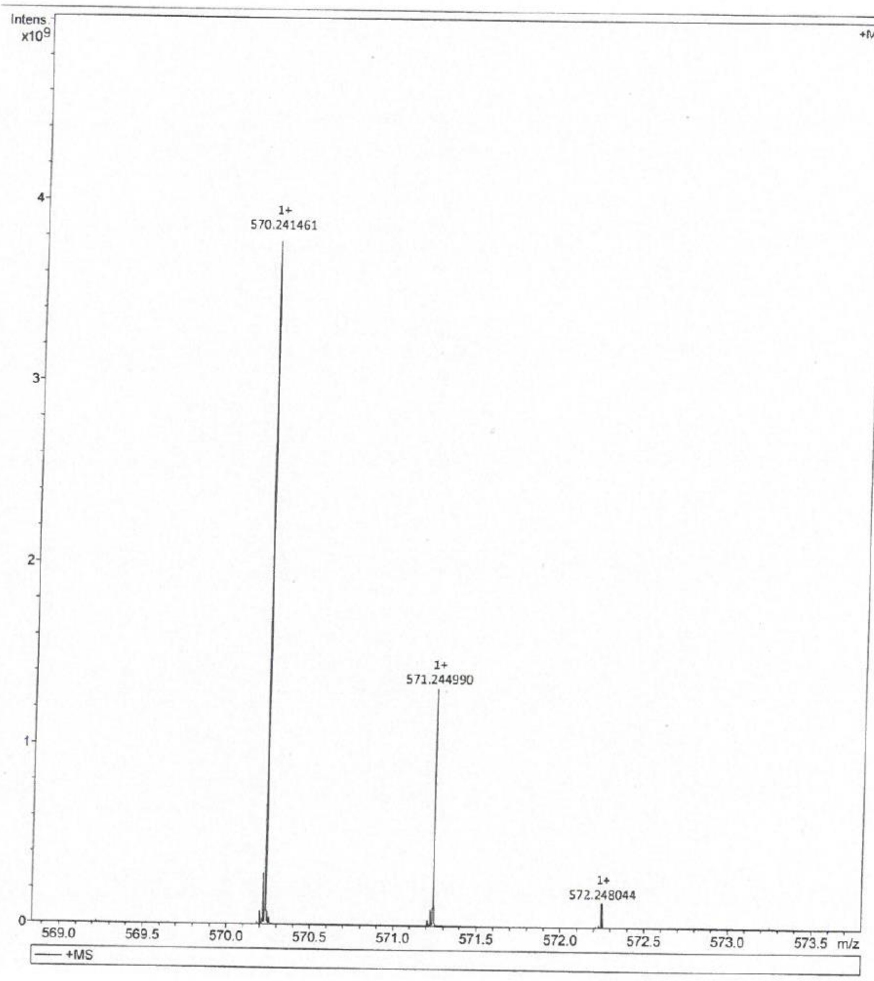

Figure SP23: Mass analysis (MALDI)(Matrix:Dithranol). m/z $[M+H^{+}]^{+}$ calcd for $[C_{33}H_{33}FN_{3}O_{5}]^{+}$: 570.2399; Found: 570.2415

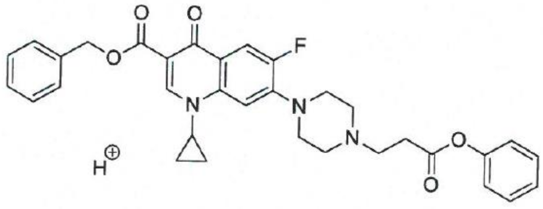

Chemical Formula: $C_{33}H_{33}FN_{3}O_{5}^{+}$
Molecular Weight: 570.6301
m/z: 570.2399 (100.0%), 571.2433 (35.7%), 572.2466 (6.2%), 571.2370 (1.1%), 572.2442 (1.0%)

Figure S47: ESI-MS of **Benzyl 1-cyclopropyl-6-fluoro-4-oxo-7-(4-(3-oxo-3-phenoxypropyl)piperazin-1-yl)-1,4-dihydroquinoline-3-carboxylate (9)**

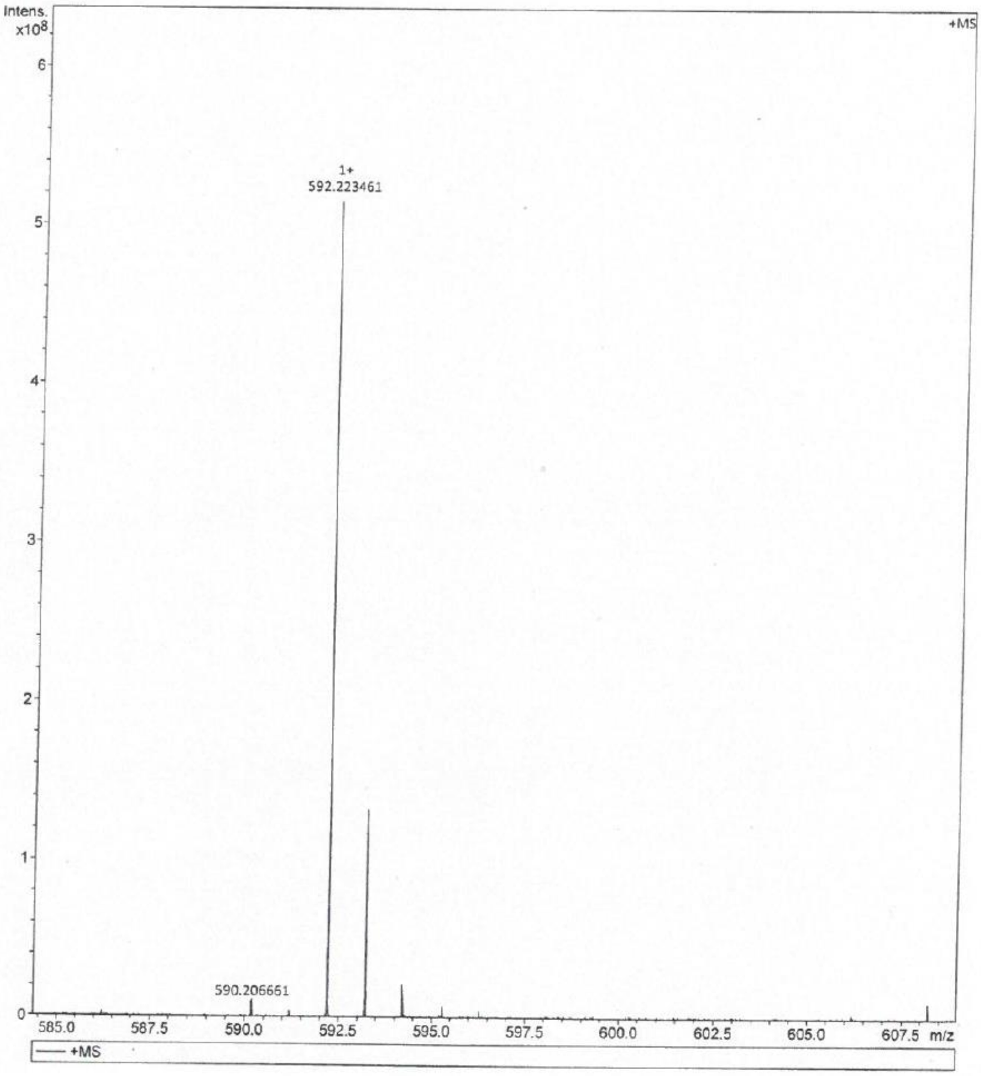

Figure SP24: Mass analysis (MALDI)(Matrix:Dithranol). m/z $[M+Na^+]^+$ calcd for $[C_{33}H_{32}FN_3NaO_5]^+$: 592.2219; Found: 592.2235

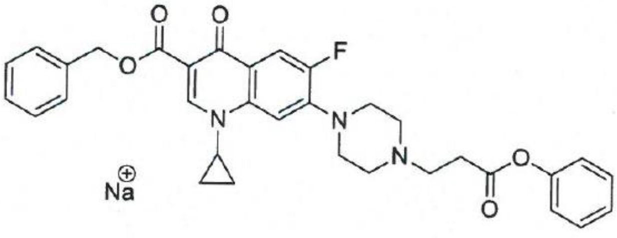

Chemical Formula: $C_{33}H_{32}FN_3NaO_5^+$

Molecular Weight: 592.6119

m/z: 592.2219 (100.0%), 593.2252 (35.7%), 594.2286 (6.2%), 593.2189 (1.1%), 594.2261 (1.0%)

Figure S48: ESI-MS of **Benzyl 1-cyclopropyl-6-fluoro-4-oxo-7-(4-(3-oxo-3-phenoxypropyl)piperazin-1-yl)-1,4-dihydroquinoline-3-carboxylate (9)**

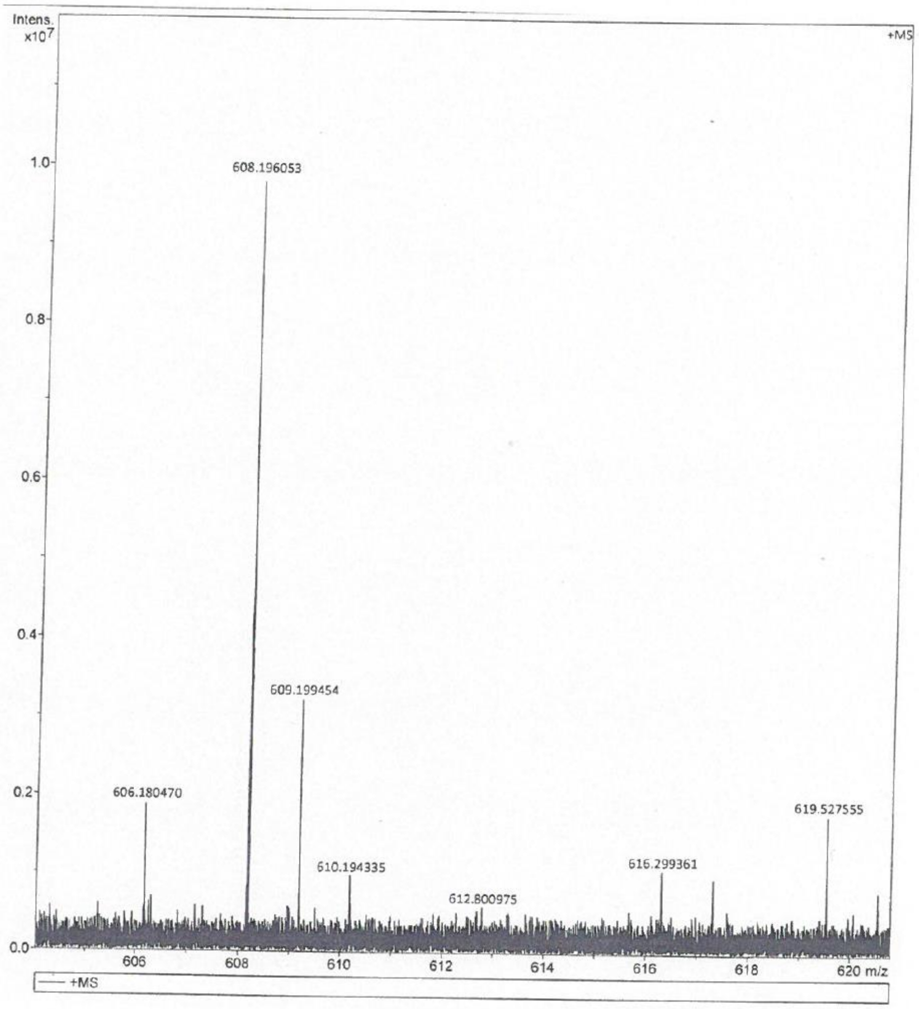

Figure SP25: Mass analysis (MALDI)(Matrix:Dithranol). m/z $[M+K^+]^+$ calcd for $[C_{33}H_{32}FKN_3O_5]^+$: 608.7204; Found: 608.1961

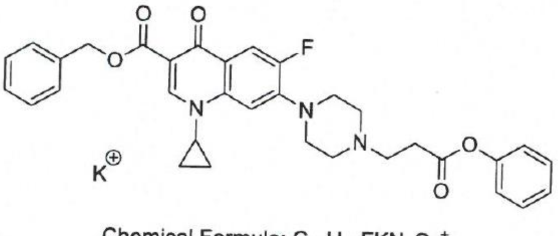

Chemical Formula: $C_{33}H_{32}FKN_3O_5^+$

Molecular Weight: 608.7204

m/z: 608.1958 (100.0%), 609.1992 (35.7%), 610.1939 (7.2%), 610.2025 (6.2%), 611.1973 (2.6%), 609.1928 (1.1%), 610.2001 (1.0%)

Figure S49: ESI-MS of **Benzyl 1-cyclopropyl-6-fluoro-4-oxo-7-(4-(3-oxo-3-phenoxypropyl)piperazin-1-yl)-1,4-dihydroquinoline-3-carboxylate (9)**

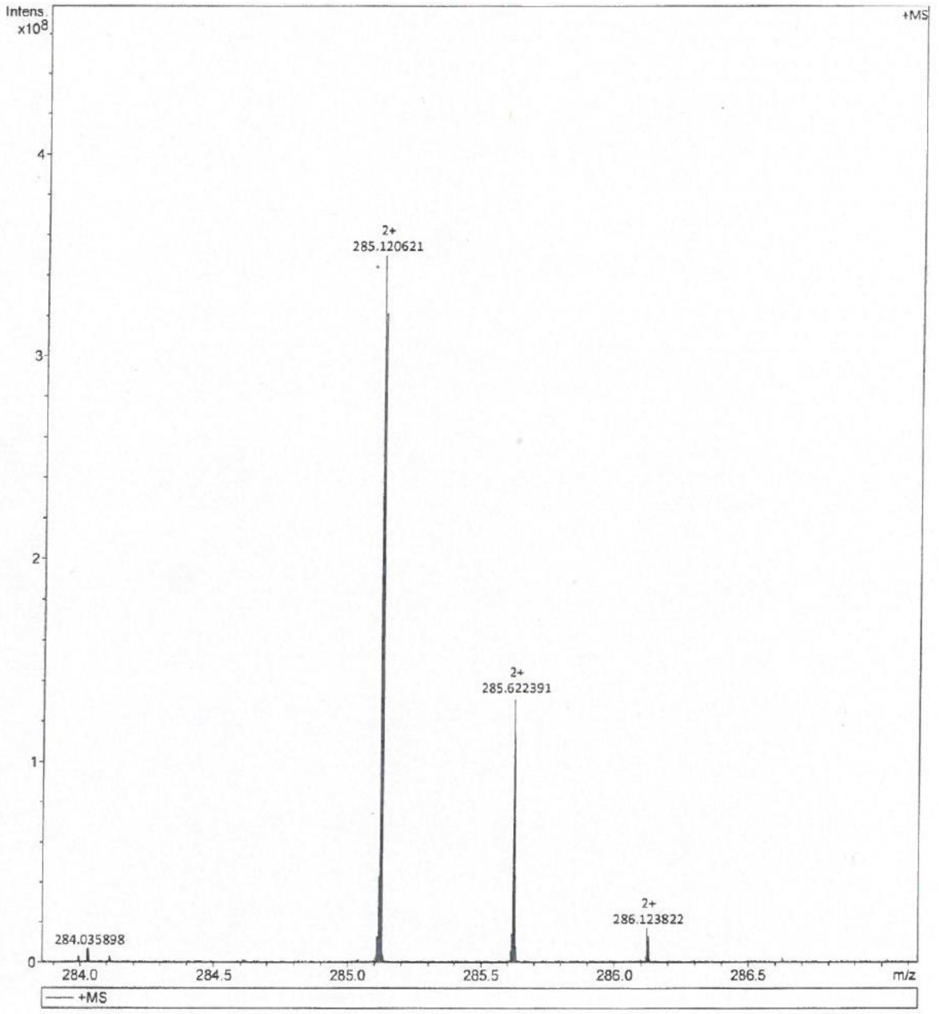

Figure SP26: Mass analysis (MALDI)(Matrix:Dithranol). m/z $[M+2H^{+}]^{2+}$ calcd for $[C_{33}H_{34}FN_{3}O_{5}]^{2+}$: 285.6236; Found: 285.6224

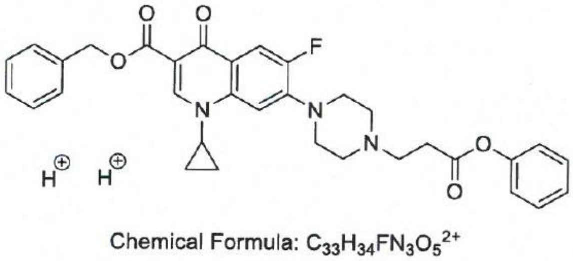

Chemical Formula: $C_{33}H_{34}FN_{3}O_{5}^{2+}$
Molecular Weight: 571.6375
m/z: 285.6236 (100.0%), 286.1253 (35.7%), 286.6270 (6.2%), 286.1221 (1.1%), 286.6257 (1.0%)

Figure S50: ^{1}H-NMR (500 MHz in DMSO-D6) of DAB-PAMAM-G0-(Cipro-Bn)$_4$ (10)

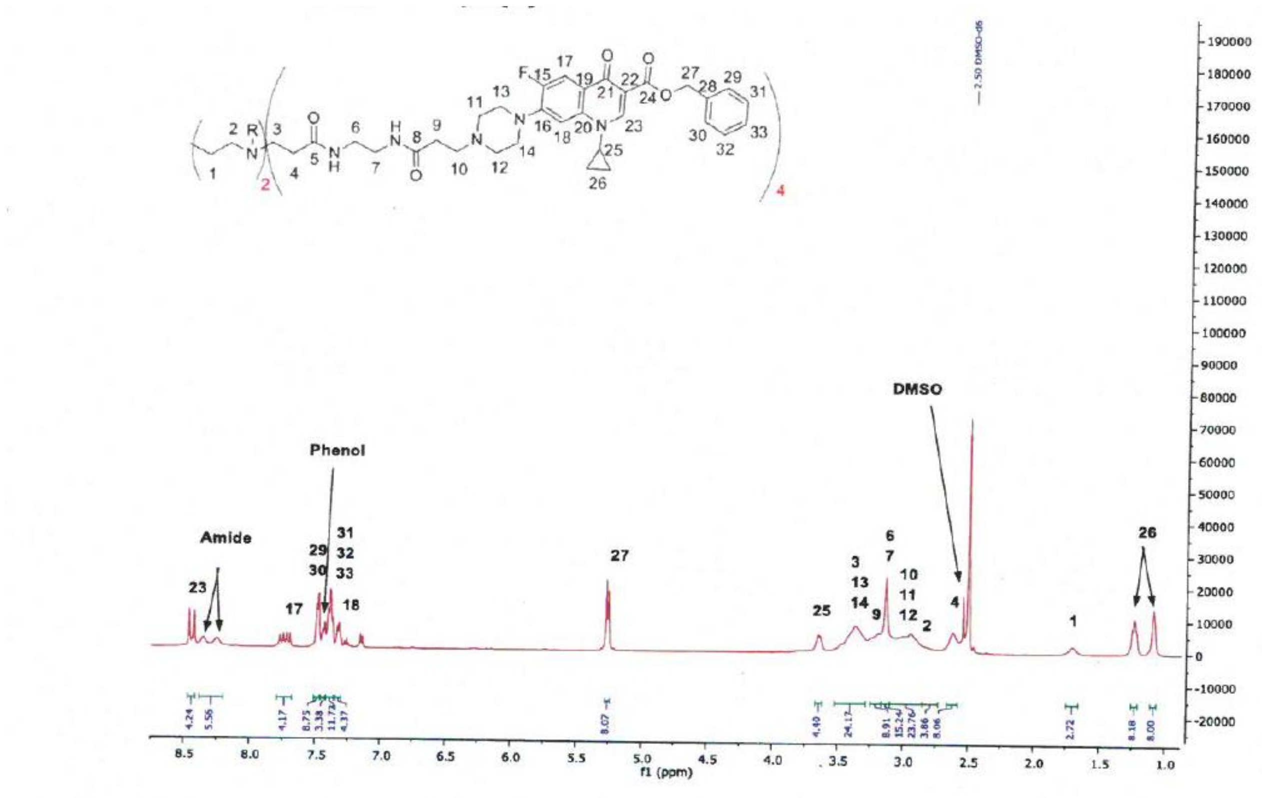

Figure S51: COSY-NMR (500 MHz in DMSO-D6) of DAB-PAMAM-G0-(Cipro-Bn)$_4$ (10)

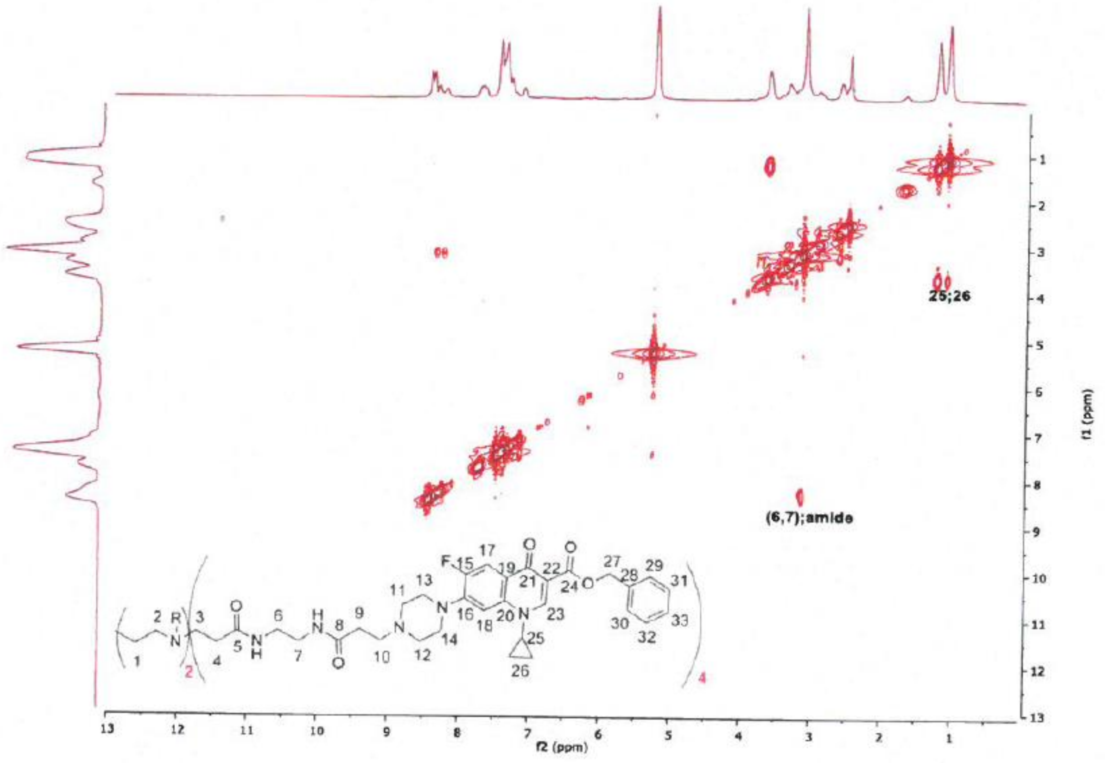

Figure S52: ^{13}C-NMR (125 MHz in DMSO-D6) of DAB-PAMAM-G0-(Cipro-Bn)$_4$ (10)

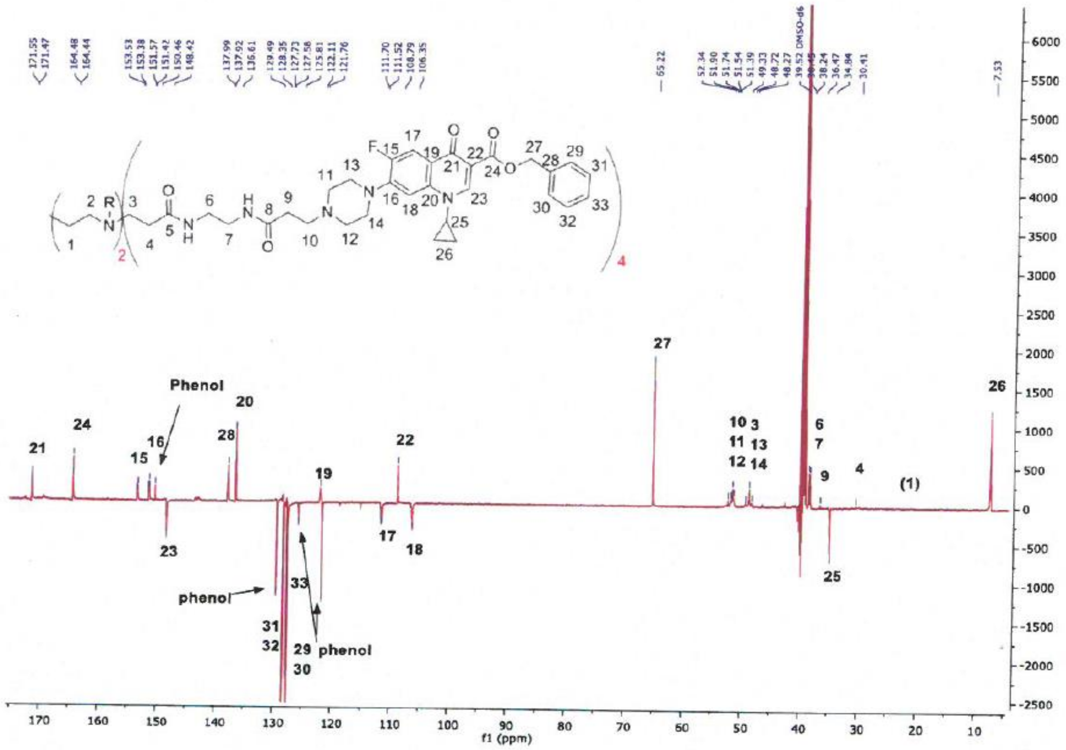

Figure S53: HSQC-NMR (125 & 500 MHz in DMSO-D6) of DAB-PAMAM-G0-(Cipro-Bn)$_4$ (10)

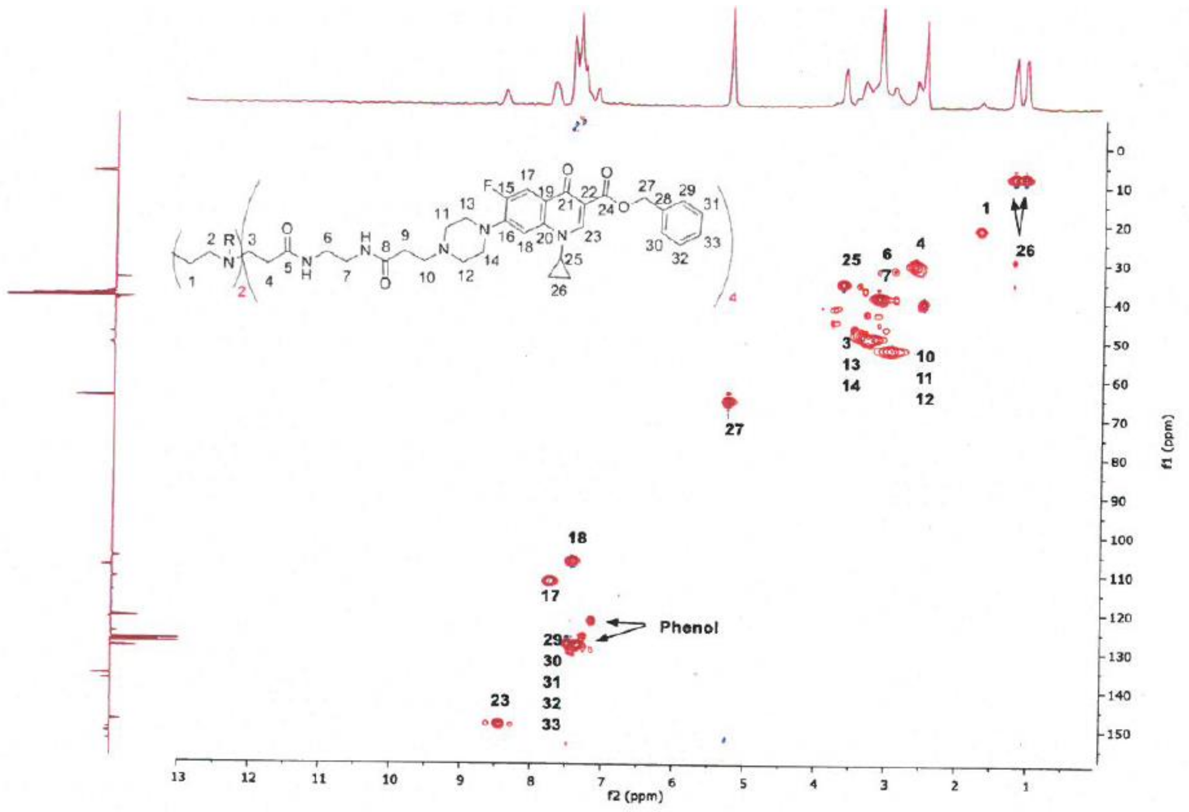

Figure S54: ^{19}F-NMR (470 MHz in DMSO-D6) of DAB-PAMAM-G0-(Cipro-Bn)$_4$ (10)

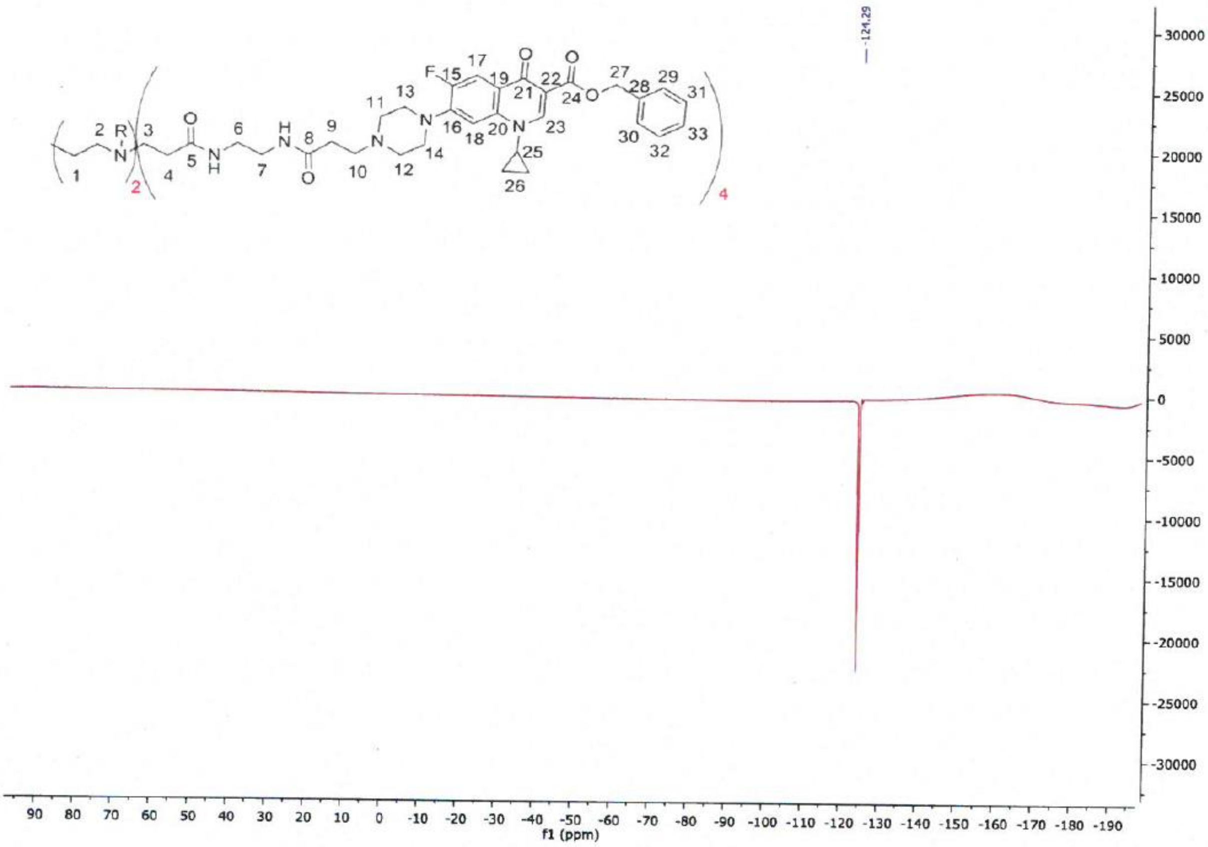

Figure S55: ESI-MS of DAB-PAMAM-G0-(Cipro-Bn)$_4$ (10)

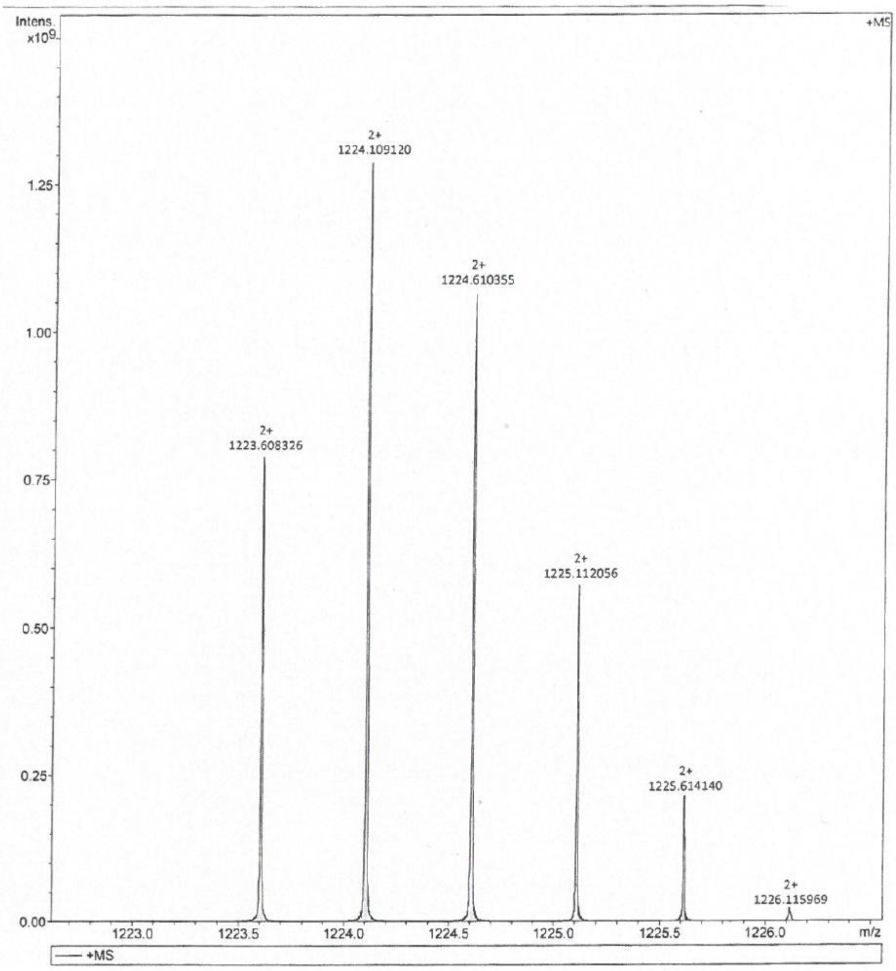

Figure SP32: Mass analysis (ESI)(MeOH+0.1%Formic acid). m/z $[M+2H^{+}]^{2+}$ calcd for $[C_{132}H_{158}F_4N_{22}O_{20}]^{2+}$: 1224.0991; Found: 1224.1091

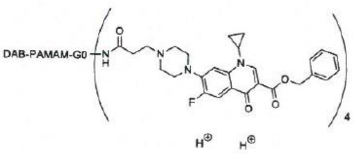

Chemical Formula: $C_{132}H_{158}F_4N_{22}O_{20}^{2+}$

Molecular Weight: 2448.7948

m/z: 1224.0991 (100.0%), 1224.6008 (70.8%), 1223.5974 (70.0%), 1225.1025 (33.2%), 1225.6041 (11.6%), 1224.5976 (8.1%), 1225.0993 (5.8%), 1224.0959 (5.7%), 1225.1012 (4.1%), 1226.1058 (3.2%), 1225.6029 (2.9%), 1224.5995 (2.9%), 1225.6010 (2.7%), 1224.6022 (1.8%), 1226.1046 (1.4%), 1225.1039 (1.3%), 1224.1006 (1.3%)

Figure S56: ESI-MS of DAB-PAMAM-G0-(Cipro-Bn)$_4$ (10)

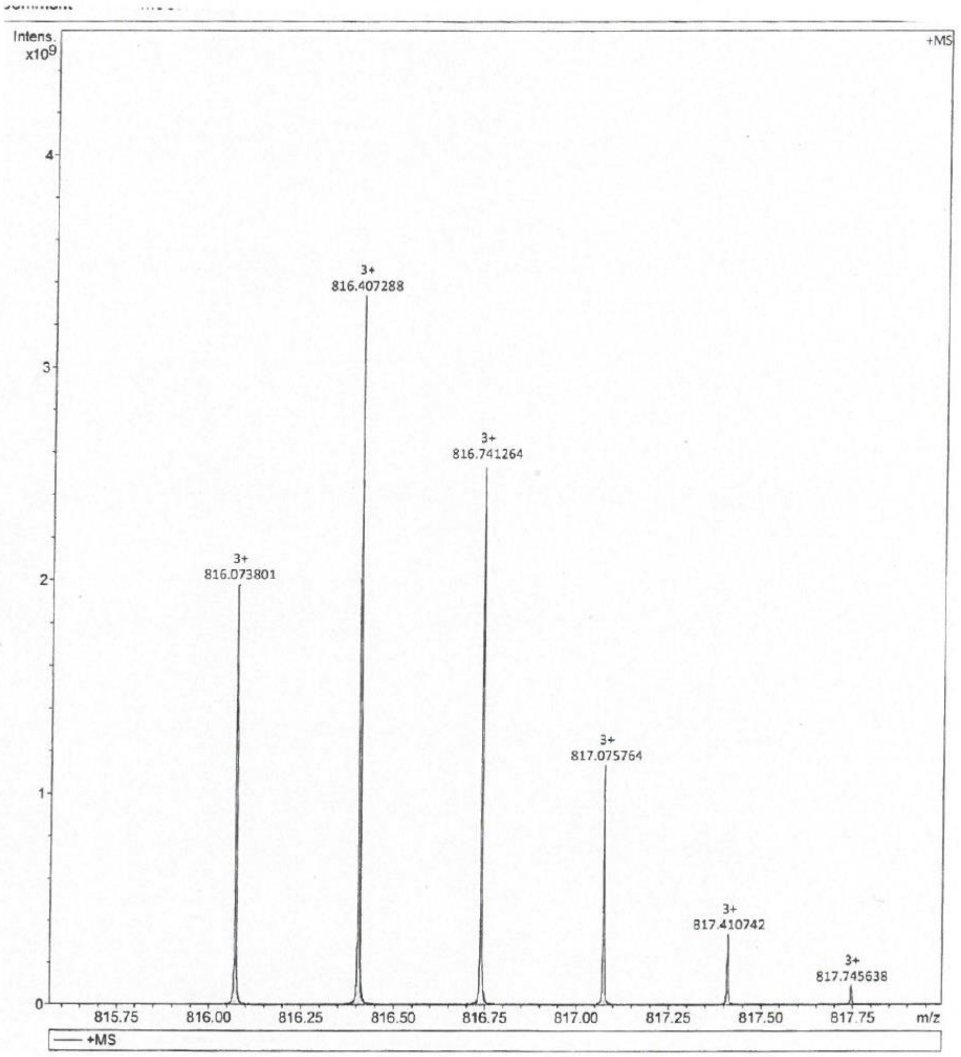

Figure SP33: Mass analysis (ESI)(MeOH+0.1%Formic acid). m/z $[M+3H^{+}]^{3+}$ calcd for $[C_{132}H_{159}F_4N_{22}O_{20}]^{3+}$: 816.4018;

Found: 816.4073

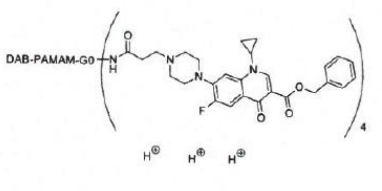

Chemical Formula: $C_{132}H_{159}F_4N_{22}O_{20}^{3+}$
Molecular Weight: 2449.8022
m/z: 816.4018 (100.0%), 816.7363 (70.8%), 816.0674 (70.0%), 817.0707 (33.2%), 817.4052 (11.6%), 816.7342 (8.1%), 817.0686 (5.8%), 816.3997 (5.7%), 817.0699 (4.1%), 817.7396 (3.2%), 817.4044 (2.9%), 816.7355 (2.9%), 817.4031 (2.7%), 816.7372 (1.8%), 817.7388 (1.4%), 817.0717 (1.3%), 816.4028 (1.3%)

Figure S57: ESI-MS of DAB-PAMAM-G0-(Cipro-Bn)$_4$ (10)

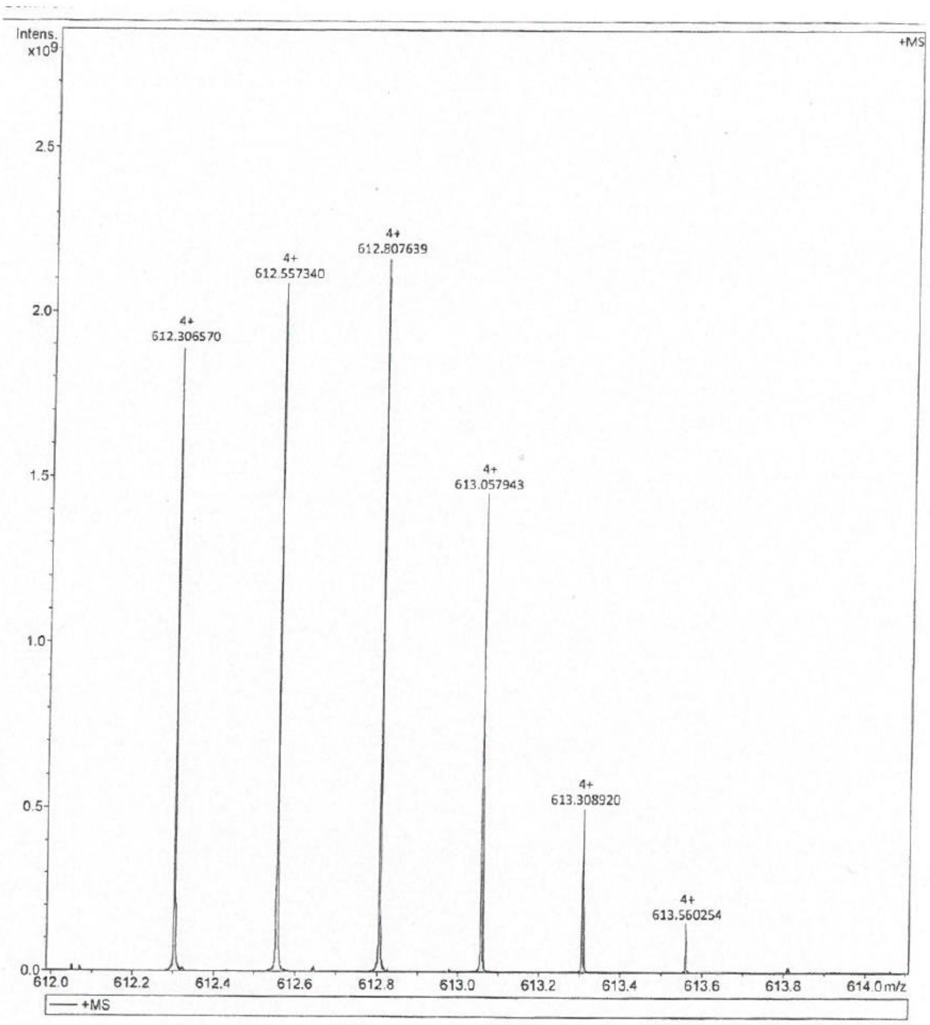

Figure SP34: Mass analysis (ESI)(MeOH+0.1%Formic acid). m/z $[M+4H^{+}]^{4+}$ calcd for $[C_{132}H_{160}F_4N_{22}O_{20}]^{4+}$: 612.5532; Found: 612.5573

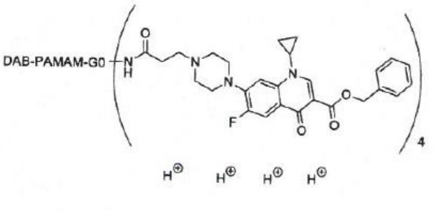

Chemical Formula: $C_{132}H_{160}F_4N_{22}O_{20}{}^{4+}$

Molecular Weight: 2450.8096

m/z: 612.5532 (100.0%), 612.8040 (70.8%), 612.3023 (70.0%), 613.0549 (33.2%), 613.3057 (11.6%), 612.8024 (8.1%), 613.0533 (5.8%), 612.5516 (5.7%), 613.0542 (4.1%), 613.5565 (3.2%), 613.3051 (2.9%), 612.8034 (2.9%), 613.3041 (2.7%), 612.8048 (1.8%), 613.5559 (1.4%), 613.0556 (1.3%), 612.5539 (1.3%)
